# Overview of Magnetic Hydrogel Fabrication, Its Basic Characteristics, and Potential Uses in Biomedical Engineering

**DOI:** 10.3390/bioengineering12111142

**Published:** 2025-10-22

**Authors:** Udit Narayan Sharma, Serge Ostrovidov, Sudipto Datta, Hirokazu Kaji

**Affiliations:** 1 Department of Polymer and Process and Engineering, Indian Institute of Technology, Roorkee 247667, Uttarakhand, India; 2 Laboratory for Biomaterials and Bioengineering (LBB), Department of Diagnostic and Therapeutic Systems Engineering, Institute of Integrated Research (IIR), Institute of Science Tokyo, Tokyo 101-0062, Japan; 3 Department of Material Science Engineering, Indian Institute of Science, Bangalore 560012, Karnataka, India; sudiptodatta1990@gmail.com

**Keywords:** magnetic hydrogels, hyperthermia, tissue engineering, magnetic field, drug delivery, MRI

## Abstract

Magnetic hydrogels are stimulus-responsive hydrogels with rapid response when placed in a magnetic field. Their properties include those of conventional hydrogels such as biocompatibility, viscoelasticity, and a high content of water, with the addition of magnetic actuation, magnetothermal conductivity, and magnetic resonance conferred by the magnetic particles. Their use in the biomedical field is constantly growing, with various applications such as drug delivery, hyperthermia treatment, theranostic, and tissue engineering. Since the research field of magnetic hydrogels is very dynamic, it is important to review the literature regularly to highlight the most recent insights of the field. In this review, we focused on the latest advances of magnetic hydrogels and give a large overview on their types, fabrication, properties, and applications in hyperthermia, drug delivery, wound healing, MRI, sensors, and tissue engineering (neural, cartilage, bone, and cardiac tissues). We concluded this review with challenges and future developments of magnetic hydrogels.

## 1. Introduction

The term ‘hydrogel’ first emerged in the academic literature as early as 1894 [1]. However, at that time, it was used for colloidal gels synthesized from inorganic salts of specific metals. Subsequently, the terminology “hydrogel” has evolved to characterize a three-dimensional (3D) network of hydrophilic polymers and gums, developed through physical or chemical crosslinking methodologies, which swells in water but does not dissolve due to the entanglements of the polymer chains [2]. The swelling property of hydrogels is attributed to their very high thermodynamic activity toward the solvent used. In recent years, this swelling characteristic, combined with the high versatility and the high tunability of hydrogels’ properties, has induced important research and developments of hydrogels, as well as their exploitation [3]. A hydrogel has its network created through covalent bonds or noncovalent forces [4]. Noncovalent interactions are mostly in the form of physical entanglements, hydrogen bonds, van der Waal forces, and aromatic, electrostatic, and coordination bonding.

The classification of hydrogels is based on their ability to respond to external stimuli, and they are divided into the following two types: static and dynamic hydrogels [4]. Static hydrogels are crosslinked through rigid covalent bonds, and their physical and chemical characteristics are barely changed regardless of the surrounding environment [4]. On the contrary, dynamic hydrogels respond to a variety of external stimuli (e.g., pressure, strain, temperature, light, pH, ions, magnetic field) and can exhibit features like self-repairing, self-shaping, or behavioral remodeling capacity [4]. Since these dynamic hydrogels demonstrate an immediate reaction to changes in their environment, they are also categorized as smart hydrogels [5]. Several investigations have been conducted on smart hydrogels regarding nanotechnology applications, drug delivery systems, and tissue engineering over the last decades [6]. However, the prolonged response latency and inadequately regulated architectures of these stimuli-responsive biomaterials constitute the two primary limitations [6].

**Figure 1:**
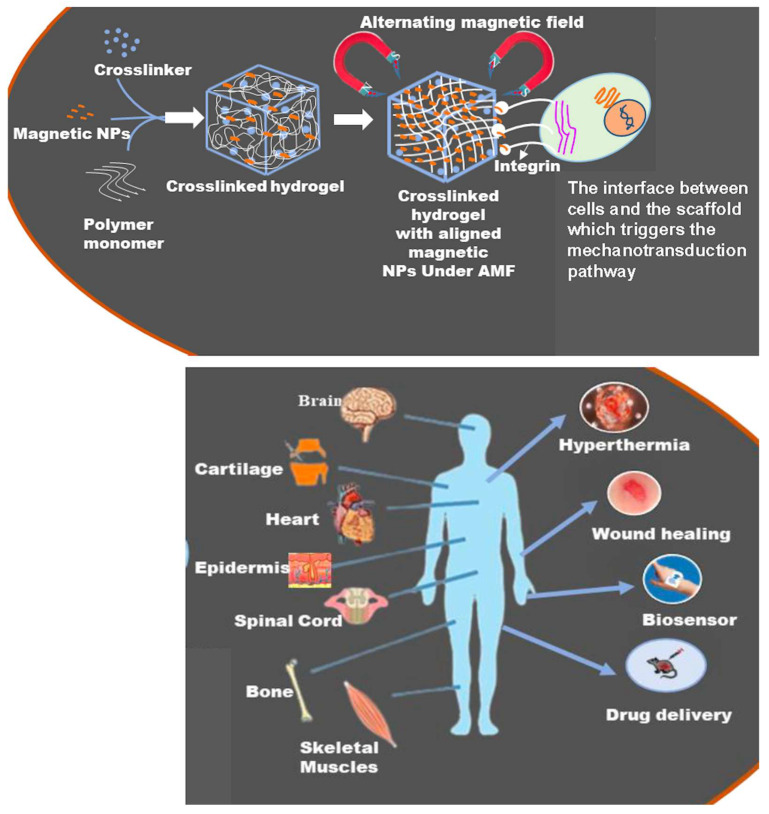
Schematic showing the synthesis of MHs and their applications.

Recently, magnetically responsive hydrogels (MHs), classified as an innovative category of smart hydrogels, have been used within the biomedical domain to improve the biological functionalities of cells, tissues, and organs (Figure 1). This is due to their capacity to respond to an externally applied magnetic field, thereby facilitating structural functionalities that enable the remote modulation of the physical, biochemical, and mechanical properties of the microenvironment surrounding the cells, tissues, or organs [6,7,8]. Thus, researchers have noted that MHs can serve as superior drug delivery and site-specific carriers. For instance, Gao et al. fabricated an MH with ferromagnetic vortex-domain iron oxide nanorings, which have superior heat induction compared to conventional super paramagnetic iron oxide nanoparticles (SPIONs) under an alternating magnetic field (AMF), and reported effective reduction in local breast tumor recurrence size under AMF activation [9]. In another study, Manjua et al. developed a magnetic responsive polyvinyl alcohol (PVA) hydrogel that could control the adsorption and release of protein via cyclic ON/OFF magnetic field activation, which could be useful in tissue engineering, drug delivery, and biosensor systems [10]. Moreover, the use of a biocomposite of a self-healing chitosan–alginate hydrogel with magnetic gelatin microspheres loaded with the anticancer drug 5-fluorouracil (5-Fu) showed efficient sustained drug delivery in vitro [11]. MHs have been investigated on various grounds, evaluating their efficiency in remote-controlled drug and cell delivery [12], bioseparation [13], magnetic resonance imaging [14], adsorption/separation (like wastewater treatment) [15], and other medical and environmental applications [16]. Thus, to assess the efficiency of chemothermal synergistic therapy for bone tumor treatment based on AMF, Hu et al. fabricated injectable doxorubicin (DOX)-encapsulated magnetic alginate hydrogel (DOX@MAH) [17]. Furthermore, Chen et al. conceived and engineered a biodegradable magnetic hydrogel robot (BMHR) which exhibits the following four stable operational modalities: tumbling mode, precession mode, spinning-XY mode, and spinning-Z mode. Due to its ability to transition smoothly between the different motion modes, the BMHR showed great adaptability in a complex environment and remarkable efficiency in transporting intended cargo [18]. In another study, Zhang et al. developed a wireless and passive flexible magnetic strain sensor using gelatin methacrylate (GelMA)/Fe_3_O_4_ magnetic hydrogel. In this work, the authors provided a demonstration of how magnetic sensing can be used for biomechanical monitoring, and proposed ideas concerning feasible wireless and passive implantable devices [19]. Furthermore, to induce controlled floating cell aggregation, Ishihara et al. fabricated cationic magnetic hydrogel microparticles that adsorbed to cells and assessed the effect of an external magnetic field on cell clustering and cellular function [20]. Singh et al. fabricated a dual-responsive MH nanocomposite with high pH sensitivity using acrylamide (AA) and vinyl sulfonic acid (VSA) monomers, which underwent polymerization via the Free Radical Polymerization technique. In this process, the monomers were blended in an optimal stoichiometric ratio, and subsequently, synthetized Fe_3_O_4_(OH)x nanoparticles were incorporated prior to the polymerization. The MH nanocomposite displayed swelling (pH 7)/deswelling (pH 4.1) behavior under pH variations [21]. In their study, Tang et al. fabricated a hybrid structure of elastomer and magnetic poly (N-isopropyl acrylamide) hydrogel to achieve different shape-morphing structures (2D and 3D) and magnetic navigation under AMF [22].

MHs have emerged as highly promising materials due to their unique properties, such as rapid responsiveness to a magnetic field and the ability to be remotely controlled [23,24]. They overcome the limitations of conventional static hydrogels and enlarge the group of dynamic hydrogels, adding new capacities and allowing for new applications in targeted therapies, including hyperthermia, drug delivery, wound healing, MRI, sensors, and tissue engineering [25,26]. Especially in tissue engineering, MHs allow for better control of cell guidance, release of bioactive factors, and tissue maturation. However, some challenges still need to be overcome for MHs to reach clinical applications. These include the inhomogeneous distribution of magnetic nanoparticles (MNPs) into hydrogels, their potential cytotoxicity, and the need for precise control of MNPs properties [27]. This review aims to provide a comprehensive summary of recent advancements in magnetic-responsive hydrogels, focusing on current strategies for their fabrication and their latest biomedical applications such as drug delivery, hyperthermia treatment, Magnetic Resonance Imaging, wound repair, biosensing, and tissue engineering (neural, cartilage, bone, cardiac tissues). Finally, we discuss the existing challenges and future directions for the development of magnetic hydrogels.

## 2. Fabrication and Characteristics of MHs

### 2.1. Strategies for Fabrication of Magnetic Hydrogels with Homogeneous Structure

**Figure 2:**
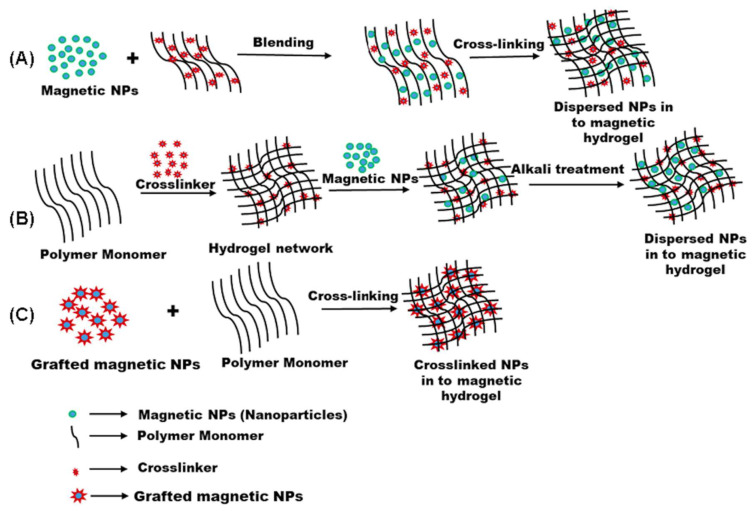
Overview of the principal methods used in the fabrication of MHs: (**A**) the blending technique, where MNPs are amalgamated with a precursor hydrogel solution at a designated molar ratio followed by crosslinking; (**B**) the in situ synthesis approach, where MNPs are generated through in situ precipitation within the polymer hydrogel network subsequent to crosslinking; and (**C**) the grafting-onto technique, which involves the introduction of multiple functional groups on the MNPs that will serve as crosslinkers.

An MH comprises a polymer matrix in which the magnetic part is incorporated. Most commonly, superparamagnetic and biocompatible iron oxide-based magnetic nanoparticles (MNPs) like maghemite (γ-Fe_2_O_3_), magnetite (Fe_3_O_4_), manganese ferrite (MnFe_2_O_4_), and cobalt ferrite (CoFe_2_O_4_) are introduced into the polymer matrix to synthesize MHs [28,29]. Magnetite, Fe_3_O_4_, is a Fe^2+^-defective spinel containing one type of Fe(II) site and two independent types of Fe(III) sites, of which the former accounts for one-third and the latter accounts for two-thirds. The phenomenon of intervalence charge transfer occurring between Fe^2+^ and Fe^3+^ results in absorption within both the ultraviolet–visible spectral domain and the infrared spectral domain, contributing to the manifestation of a black appearance [30].The factors that influence the properties of MHs are (i) the type of hydrogel used and the type of MNPs incorporated, (ii) size distribution and distribution pattern of MNPs in the hydrogel network, and (iii) concentration of hydrogel, MNPs, and other components used [31]. The main fabrication methods of MHs include the in situ precipitation technique, the blending approach, and the grafting strategy (Figure 2) [32].

In the blending method (Figure 2A), the hydrogels and the MNPs are prepared separately. The MNPs are often prepared using a precipitation method, and they are instantly mixed with the solubilized hydrogel polymer. A sonication step is used to enhance the dispersion of MNPs in the polymer solution. Then, the polymer solution with the MNPs is crosslinked, forming the MH. Due to the separate synthesis of MNPs, the method of preparation of MH provides a way to introduce MNPs with the same size in the polymer solution. However, obtaining a homogenous distribution of MNPs in hydrogels is not easy, since the MNPs may agglomerate or diffuse out of the hydrogels when the MHs are put in aqueous solution. In the in situ precipitation technique (Figure 2B), the hydrogel network is formed first by crosslinking and serves as a chemical reactor in which an inorganic salt containing iron ions will react with precipitating agents such as NaOH or NH_4_OH, resulting in the formation of MNPs, as elucidated by Haas et al. [31], entrapped in the hydrogel. Thus, after the polymer solution is crosslinked and formed into a hydrogel, the hydrogel is immersed in a solution containing Fe^2+^/Fe^3+^ ions; in this case, the ions can penetrate the structure of the hydrogels and distribute into it. Then, hydrogels containing Fe^2+^/Fe^3+^ ions are treated with alkali such as NaOH or NH_4_OH and the magnetite precipitates [31]. The in situ precipitation technique allows the networks of hydrogels to have an optimal distribution of Fe_3_O_4_ nanoparticles. However, this approach is only possible when the formed hydrogels have stable networks that are not affected or decomposed by the addition of alkali solutions during MNP synthesis [33]. Furthermore, the encapsulation of cells during MH formation is not possible due to the use of alkali solutions. In the context of the blending and in situ precipitation methodologies, the occurrence of chemical interactions between the MNPs and the polymeric hydrogel matrix is precluded. Consequently, the distribution of MNPs within the hydrogels cannot be clearly defined. In contrast, in the grafting technique, the incorporation of MNPs is executed in a particularly unique manner, and covalent immobilization of MNPs within the hydrogel matrix is realized (Figure 2C). Likewise, analogous to the blending method, both hydrogels and MNPs are synthesized separately in the grafting approach. In the preparation of MNPs, the functional groups are incorporated into the surface of MNPs at the co-precipitation step, and the stability of the MH network is attributed to the covalent bonds formed between the MNPs and the polymeric chains. Regarding the grafting methodology, it is observed that this approach is used less frequently with natural polymer, compared to the blending or in situ precipitation techniques, due to the insufficient availability of reactive sites on natural polymers for such grafting modifications [34].

### 2.2. Strategies for Fabrication of Magnetic Hydrogels with Ordered Structure

**Figure 3:**
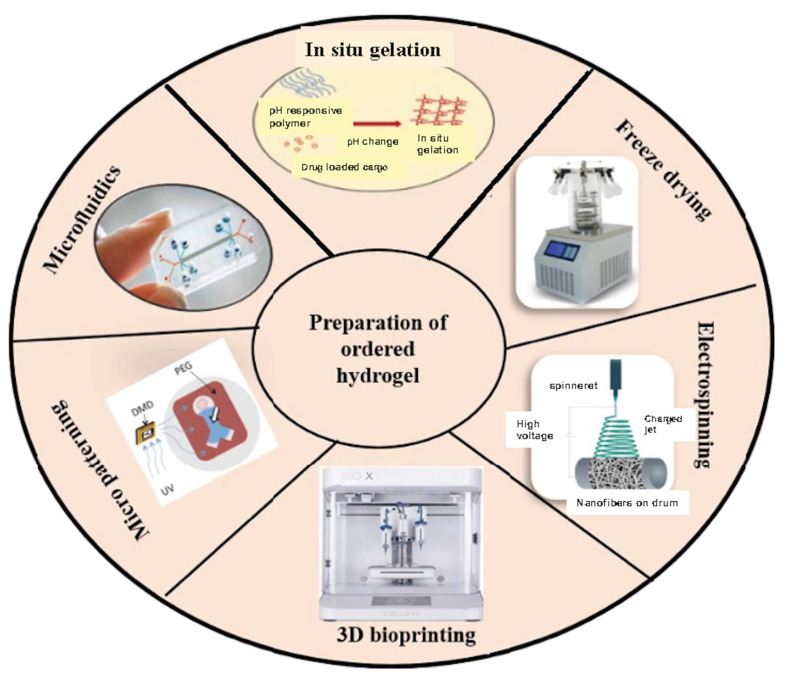
Schematic showing the different methods used for the fabrication of MHs with well-ordered architecture.

Conventional MHs have homogeneous structures with a homogeneous distribution of MNPs. However, this isotropy limits their applications with biological tissues, which often have a hierarchical organization. Therefore, researchers also develop anisotropic MHs with MNPs distributed in the hydrogels in an orderly manner to enhance their functionalities and interactions with biological anisotropic tissues [7]. As shown in Figure 3, many techniques have been used to fabricate MHs with an ordered structure, including 3D bioprinting, microfluidics, and magnetic-field-induced assembly. Over the years and based on the biomedical application required, different techniques have been used to obtain hydrogels with different properties and characteristics [7,35].

Magnetic-field-induced assembly is an easy method to fabricate MHs with an ordered structure. The magnetic NPs (ferromagnetic, paramagnetic, superparamagnetic) align along the magnetic field and form a chain-like or column-like assembly inside the hydrogel due to magnetic dipolar interactions, and once the hydrogel is crosslinked (heat, light, UV, chemical), the magnetic structure is fixed [36,37,38]. Interestingly, Fan et al. showed that the use of a rotating magnetic field allowed for the gathering of the Fe_3_O_4_ NPs in the shape of disks (from few micrometers to 23 μm) in a polyacrylamide hydrogel [39]. These MHs with an oriented structure find applications in tissue engineering [40,41], drug delivery [42,43], hyperthermia [22,44], and others (e.g., photonic, actuation).

The microfluidic technique is a powerful method for the generation of polymeric beads (using T junction, co-flows, or flow focusing devices), microgels, and MHs [45]. By precisely controlling the flow in the channel, the number, size, morphology, and structure of the microparticles can be defined and obtained. Thus, MHs with various shapes have been fabricated, such as fiber-like, Janus beads, multi-compartment beads, and peanut-like [46,47]. Interestingly, recently, more and more complex nano/micro-polymeric particles have been fabricated [48], including magnetic spring microfibers [49] and magnetic structural color hydrogels for photonic crystal and biomimetic applications [50].

Three-dimensional printing is an additive manufacturing technique which allows for the layer-by-layer fabrication of complex three-dimensional structures by precise deposition of a matrix under the control of a computer, following a design made with a computer-aided design (CAD) software. When cells are mixed with the matrix and printed, the technique is named 3D bioprinting [51,52,53]. Three-dimensional printing allows for the rapid fabrication of MHs with ordered structures, complex geometries, different scales, and multiple materials [54]. Different printing techniques, such as inkjet printing (IJP), two-photon polymerization (TPP), and direct ink writing (DIW), have been used for the fabrication of MHs. An interesting example is given by Siminska-Stanny et al., who used inks with three different concentrations (0, 10, and 20%) of magnetic fillers and 4D printed by extrusion graded MHs that were further crosslinked with calcium ion solution [55].

Another method for preparing isotropic and anisotropic MHs is freeze-drying. The fabrication of freeze-dried MHs will depend on certain factors (e.g., the temperature, the freezing time, and the melting time) that affect the physical properties of the hydrogel (e.g., the microstructure, the swelling capacity, and the degradation) [56]. For instance, we may obtain scaffolds with an average pore size of 325 µm to 85 µm when the freezing temperature used is −10 °C and obtain an average pore size of 85 µm when the freezing temperature used is −70 °C. This phenomenon is likely to be caused by temperature leading to a larger ice crystal within the scaffold [57]. Moreover, the freeze-drying method is often used to introduce pores of different sizes in the hydrogel, which will enhance the diffusion rate of encapsulated active molecules within the scaffolds [58]. The following table (Table 1) reviews some advantages and disadvantages of the different fabrication strategies.

**Figure 4:**
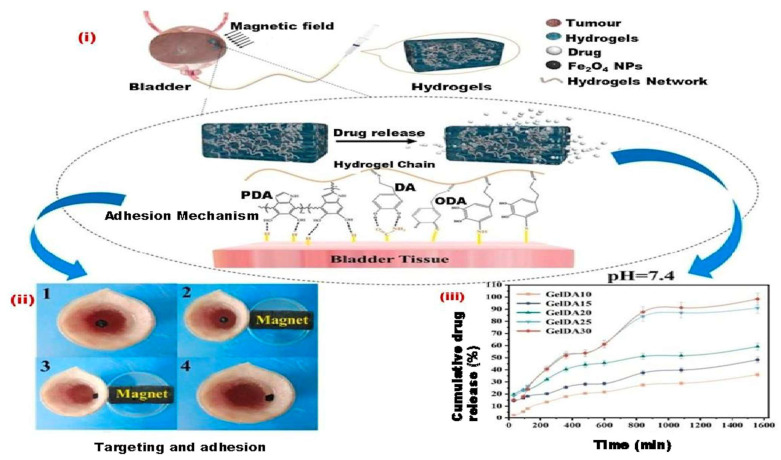
(**i**) Schematic representation of a hydrogel-based approach for bladder cancer treatment. (**ii**) Mechanism of gel formation. Targeting capability and tissue adhesion properties of the GelDA hydrogel. (**iii**) Release profile and corresponding fitting curves of bovine serum proteins at pH 7.4. Reproduced with permission from [69]. Copyright 2024, Elsevier B.V.

The freeze-drying technique has been applied to the fabrication of MHs. Thus, Wu et al. fabricated anisotropic PVA hydrogels with carbonyl iron particles, forming aligned chains in the hydrogel which improved the compression property and magnetorheological effects [68]. Chen et al. designed innovative hydrogels for bladder cancer treatment by developing a targeted drug-delivery system. As illustrated in Figure 4, these hydrogels were enriched with catechol moieties to enhance their adhesion properties. Additionally, iron tetraoxide nanoparticles (Fe_3_O_4_ NPs), commonly utilized in biomedical research, were incorporated to provide targeting capability. The hydrogels were synthesized through a simple and cost-effective process using readily available materials. Their micromorphology and potential crosslinking were thoroughly characterized. This novel self-adhesive targeted drug-delivery hydrogel presents a promising approach to enhancing drug therapy efficiency for bladder cancer while reducing patient discomfort during treatment [69].

**Figure 5:**
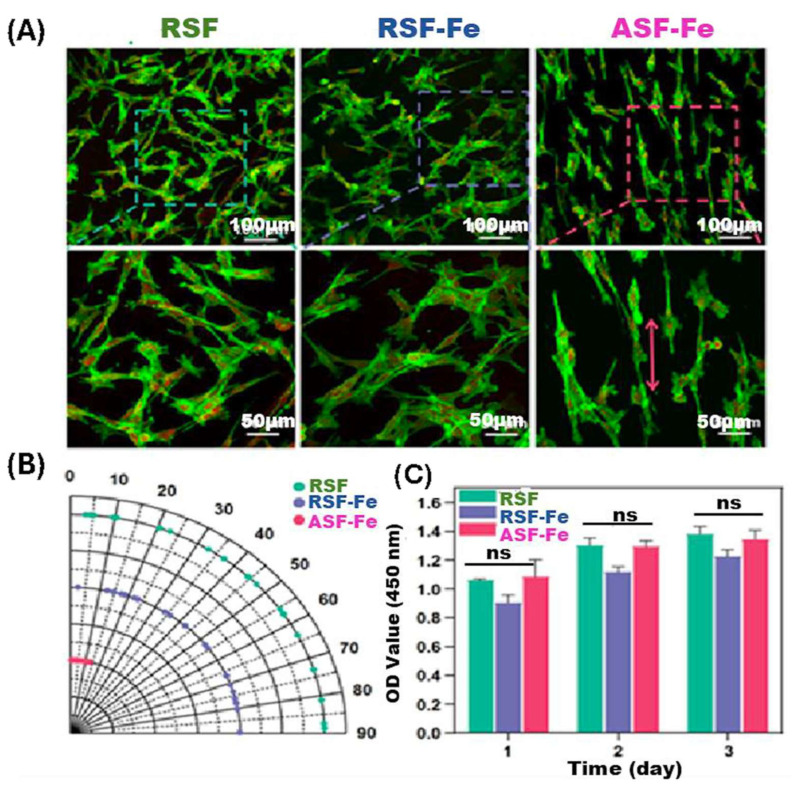
Representative confocal laser micrographs (**A**) showing C2C12 cells cultured on RSF, RSF-Fe, and ASF-Fe nanofibers, along with their orientation angle (**B**). Additionally, the MTS assay results (**C**) depict mesenchymal stem cell (MSC) proliferation. The results of cell proliferation analysis show no significant differences (ns). Reproduced with permission from [76]. Copyright 2024, American Chemical Society.

In electrospinning, the polymer is injected in a charged spinneret due to a high electrical potential (15–30 kV) applied between the spinneret and the metallic collector. When the electrical potential overcomes the surface tension, a polymeric jet is ejected from the spinneret and undergoes elongation and solvent evaporation, forming a nanofiber [70,71,72]. The genesis of nanofibers, along with the specific nanofiber morphology, will be determined by the polymeric concentration, the electrical conductivity, the viscosity, the molecular weight, the solvent volatility, and the structural composition of the polymer solution [73]. Electrospinning has important applications in tissue engineering and drug delivery [74], and nanofibers with MHs have been fabricated. For example, Sousa et al. fabricated PCL/gelatin nanofibers incorporating SPIONs for neural tissue regeneration [75]. In skeletal muscle tissue engineering, anisotropic materials that replicate natural tissue architecture hold significant potential. Electrospun scaffolds designed to mimic the extracellular matrix’s fibrillar structure are frequently used. Silk fibroin (SF) has gained attention in tissue engineering due to its exceptional biocompatibility, mechanical resilience, and biodegradability. Thus, in their study, Yang et al. developed a simple yet effective approach to fabricate directional tissue scaffolds using SF. By integrating a magnetic field collection system and incorporating Fe_3_O_4_ nanoparticles into the spinning solution, they successfully generated well-aligned silk nanofiber scaffolds. These aligned fibers not only enhanced the scaffold orientation and mechanical strength, but also demonstrated magnetic responsiveness. Furthermore, the aligned SF scaffolds facilitated mesenchymal stem cell adhesion, proliferation, and differentiation along the fiber direction. Myoblast C2C12 cells cultured on these scaffolds exhibited directional growth (Figure 5) [76].

**Figure 6:**
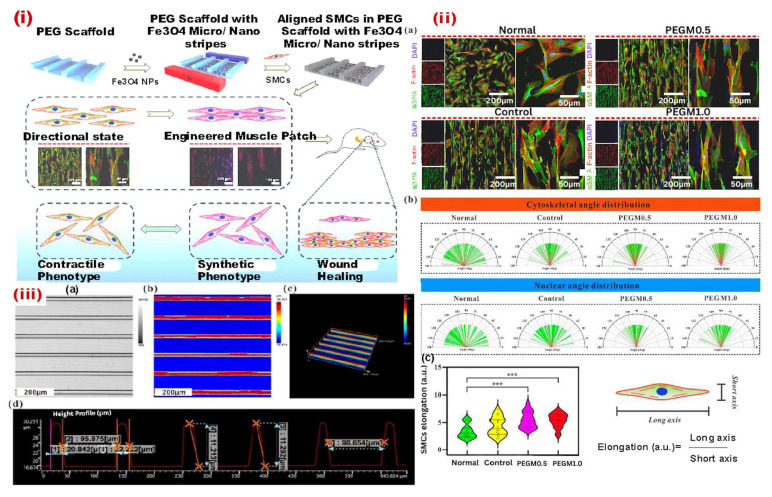
(**i**) A schematic representation illustrating the fabrication of the scaffold and the induction of cellular alignment for engineered muscle using a micro-channeled PEG scaffold with integrated magnetic Fe_3_O_4_, designed to enhance esophageal muscle regeneration. (**ii**) Assessment of cell orientation on various substrates: (**a**) Immunofluorescence (IF) staining images depicting the orientation of SMCs cultured on different substrates, including standard culture plates (normal), PEG scaffold (control), PEGM0.5, and PEGM1.0, after 24 h. Blue fluorescence of DAPI for nucleus, red fluorescence for F-actin, and green fluorescence for α-SMA (marker of SMC differentiation) (**b**) Statistical analysis of cytoskeletal and nuclear orientation angles. (**c**) Quantitative evaluation of SMC elongation. *** indicating *p* < 0.001. (**iii**) Characterization of the PEG scaffold: (**a**) surface morphology, (**b**) apparent topological structure, (**c**) 3D structure visualization, and (**d**) size measurement using 3D laser confocal microscopy. Reproduced with permission from [77]. Copyright 2023, The Authors (open access).

The micropatterning technique can also be used to fabricate ordered magnetic hydrogel (Figure 6). Luo et al. introduced aligned nano-ferroferric oxide (Fe_3_O_4_) assemblies onto a micropatterned poly(ethylene glycol) (PEG) hydrogel, creating micro-/nano-stripes (Figure 6i). Further enhancement with a gold coating improved cellular adhesion, orientation, and organization within these structures, effectively limiting smooth muscle cell (SMC) adhesion to the Fe_3_O_4_-patterned channels while preventing excessive attachment to the thin PEG ridges. This structural design facilitated cytoskeletal alignment and actin filament elongation, promoting the organized formation of muscle bundles and encouraging SMCs to adopt synthetic phenotypes (Figure 6ii). Muscle patches derived from these micro-/nano-stripes (Figure 6iii) were transplanted into a rat esophageal defect model, where in vivo studies confirmed their high viability and effectiveness in accelerating esophageal tissue regeneration. This approach offers a promising strategy for constructing muscle patches with precise alignment and enhanced muscle bundle formation, advancing the field of muscle tissue engineering [77].

**Figure 7:**
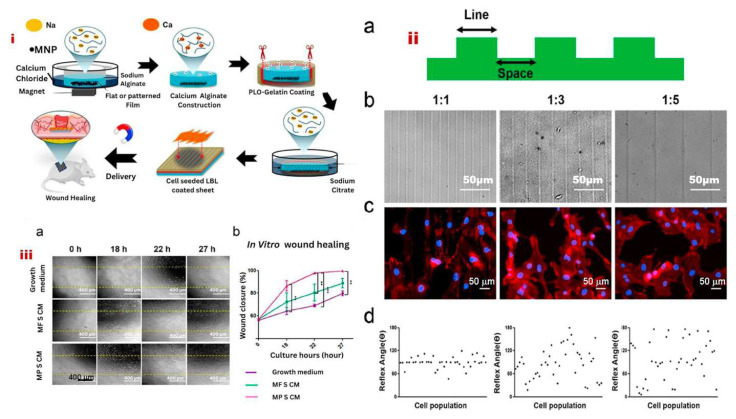
(**i**) Schematic representation illustrating the synthesis of MNP-embedded hydrogel sheets with groove patterns for wound healing applications. (**ii**) Characterization of hydrogel sheets featuring different groove patterns: (**a**) Cross-sectional view of the groove structures. (**b**) Bright-field microscopy images showcasing hydrogel sheets with groove patterns at varying line-to-space ratios: 1:1 (10:10 μm), 1:3 (10:30 μm), and 1:5 (10:50 μm). Scale bar = 50 μm.(**c**) F-actin (red)/DAPI (blue for nucleus) staining images depicting cellular adhesion and alignment on the patterned hydrogel sheets. (**d**) Quantitative analysis of cell orientation (*n* = 40), scale bar = 50 μm. (**iii**) In vitro wound healing assay: (**a**) Bright-field images capturing the wound healing process at different time points (0, 18, 22, and 27 h). (**b**) Quantitative evaluation of wound closure rates (*n* = 3), with statistical significance indicated as ** *p* < 0.001, *** *p* < 0.0001. Scale bar = 400 μm. Reproduced with permission from [78]. Copyright 2019, American Chemical Society.

Noh et al. explored the role of endothelial progenitor cells (EPCs) in promoting pro-angiogenic responses during tissue repair. EPC transplantation has gained significant attention in wound healing applications, and an optimal scaffold design that supports cell retention and function is essential for effective in situ delivery. In this study, an alginate/poly-l-ornithine/gelatin (alginate-PLO-gelatin) hydrogel sheet with a groove pattern was developed as a cell delivery platform. The topographical modification of the hydrogel surface was designed to regulate cell proliferation, alignment, and elongation. The patterned substrate facilitated morphological changes in endothelial cells (Figure 7), strengthened cell–cell interactions, and stimulated the secretion of growth factors such as platelet-derived growth factor subunit BB (PDGF-BB). Additionally, MNPs were integrated into the patterned hydrogel sheet, enabling magnetic field-assisted transfer of cell-seeded hydrogels. This innovative approach resulted in improved wound healing through efficient EPC transplantation using an MNP-embedded patterned hydrogel sheet (MPS). Ultimately, the EPC-seeded MPS enhanced vascularization and accelerated dermal wound repair [78].

### 2.3. Fundamental Characteristic of MHs

**Figure 8:**
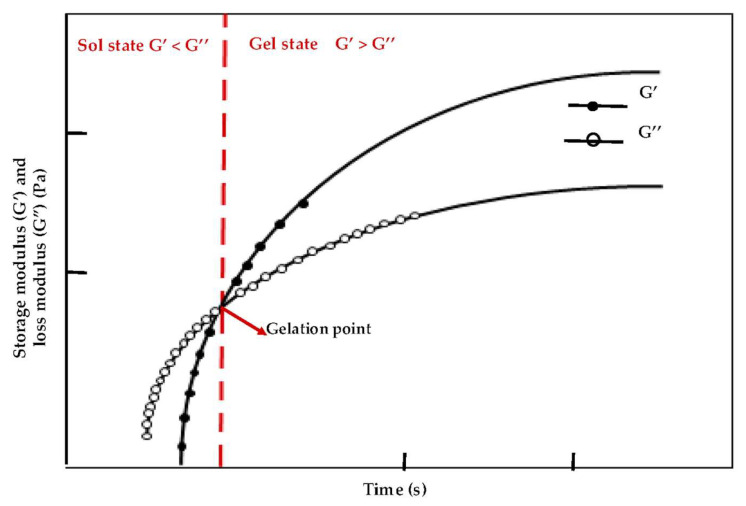
An analysis of the rheological characteristics during the sol–gel transition of a hydrogel-forming solution. Modified with permission from [79]. Copyright 2005, Elsevier Ltd.

The most incorporated characteristic that differentiates a hydrogel from other liquid media (e.g., polymer melts or solutions, suspension, and films) is the achieved storage modulus (G’) and loss modulus (G’’), which define the elasticity and the viscoelasticity of the hydrogels, respectively, as depicted in Figure 8. For instance, the frequency that is obtained from the cross of the real part of the elastic modulus (G’) and the loss modulus (G”) is deemed as the gelation point of hydrogels [79].

#### 2.3.1. Surface Properties

The surface chemistries of a material significantly account to its biocompatibility. The connection of a biological component (e.g., protein, cell, and tissue) to a hydrogel occurs on its surface, which is deemed as the primary and initial surface. Therefore, the physicochemical characteristics of the hydrogel, as well as the surface features, play a critical role in cell adhesion and proliferation. For example, some hyaluronic (HA) hydrogels may be comparatively more crystalline and, therefore, more surface-ordered, while others are comparatively more amorphous. Similarly, their surfaces can be rougher or smoother. Moreover, hydrogels can also be more hydrophilic or hydrophobic. Thus, polyvinyl alcohol (PVA) is a hydrophilic polymer, while polydimethylsiloxane (PDMS) or polyurethane (PU) are hydrophobic polymers [80]. In addition, the surface of the hydrogel may be electrically charged [81]. The hydrogel surface can also provide adhesion to cells motifs or not. Thus, to improve the cell attachment on some hydrogels like PEG hydrogels, functional groups (like Arg-Gly-Asp peptide) have been introduced onto their surfaces [82]. These different surface properties will determine the efficiency of the hydrogel surface for protein and cell adhesion, and later the cell organization and tissue formation [83,84].

#### 2.3.2. Biocompatibility

**Figure 9:**
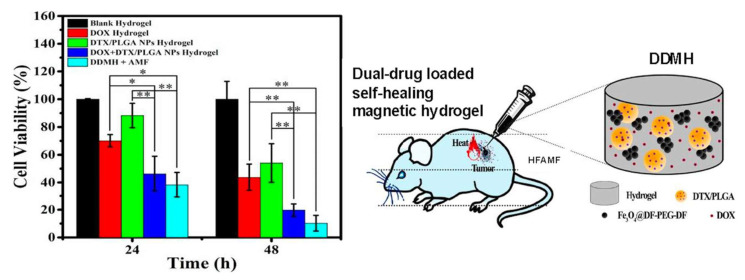
Cell viability assessments of MDA-MB-231 cells following treatment with either monotherapy or combination chemotherapy. The viability of MDA-MB-231 cells was quantified using the Cell Counting Kit 8 (CCK-8) assay. The reported values are expressed as mean ± standard deviation (SD); *n* = 5 (* *p* < 0.05, ** *p* < 0.001). Reproduced with permission from [90]. Copyright 2017, American Chemical Society.

Biocompatibility is broadly classified into two types, namely bulk biocompatibility and interfacial biocompatibility. The capacity of a material to apply load and influence the physiological and mechanical conditions of the systems it encapsulates is called bulk biocompatibility, or mechanical biocompatibility. Thus, parameters such as Young’s modulus, tensile strength, ductility, fatigue life, fretting fatigue life, wear properties, and functionalities correlate with mechanical biocompatibility [85]. In contrast, protein adsorption and cell adhesion are considered under interfacial biocompatibility and are important to understand the material’s interactions with the surrounding biological world. For the purpose of biomedical usage, biocompatibility seems to be more related to interfaced compatibility than compatibility in bulk [86,87]. Thus, blood compatibility, for example, means the ability of a material to be in a direct contact with the blood without inducing coagulation, thrombosis, and changes in blood composition or blood function. Hydrogels that are in direct contact with blood should demonstrate a good blood biocompatibility, especially hemostatic dressings [88,89]. Moreover, the histocompatibility of a material means that the material does not induce an immune response by interfering with the major histocompatibility complex (MHC). MHs are fabricated from natural (e.g., fibrin, chitosan, hyaluronic acid, and collagen) and synthetic biomaterials (e.g., PEG, PGA) used as a matrix for encapsulating the magnetic component, and this matrix must be biocompatible, as well as its degradation byproducts (Figure 9) [90].

**Figure 10:**
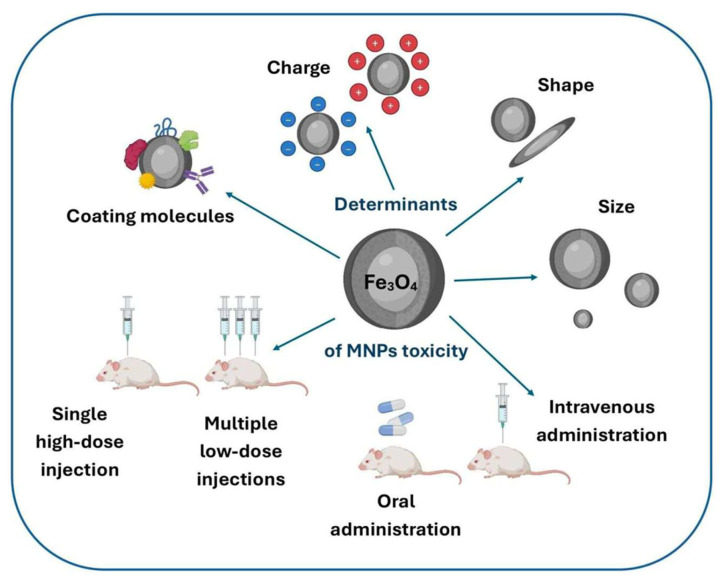
Schematic showing several factors that act on the toxicity of MNPs, and the need for testing MNPs in vivo. Reproduced with permission from [91]. Copyright 2025, The authors (open access).

In addition, although the concentration of MNPs in most MHs is often less than 1 wt%, the biosafety of MNPs is a consequence of the different MNPs released during MH degradation. The toxicity of MNPs is an important and difficult question with contradictory results. This toxicity is multifactorial, influenced by several parameters such as the MNP size, coating, surface charge, concentration, exposure time, and the type of cells or tissues exposed to MNPs (Figure 10) [91,92]. Therefore, toxicity assessment is required for each new developed nanoparticles, as well as their in vivo biodistribution and clearance.

MNPs’ toxicity is associated with the generation of reactive oxygen species (ROS) and reactive nitrogen species (RNS). In particular, ferric MNPs at substantial concentrations may increase the generation of ROS [93]. Iron is an abundant and important element in the human body, notably involved in oxygen transport by heme molecules such as hemoglobin. However, iron is usually not free in the body, but chelated to avoid uncontrolled formation via the Fenton reaction of hydroxyl radicals (HO^•^), which are highly reactive and toxic to living cells. Mitochondria that generate adenosine triphosphate (ATP) via cell respiration and the electron transport chain are particularly susceptible to dysfunction by labile iron, and ROS generation due to MNP exposure has been observed in various cells, including MCF-7, mouse primary macrophage, rat lymphocytes, human endothelial cells, hamster lung cells, osteosarcoma, and human lung A549 cells [94,95,96].

Because uncoated MNPs have a tendency to aggregate together, MNPs are often coated with polymers and small molecules to enhance their stability in aqueous media [97]. In addition, coating allows for improving the functionalization of MNPs and drug loading and delivery. However, to avoid or reduce MNPs’ toxicity, coating with polymers such as PEG and PLGA can be used [98]. Another strategy to mitigate MNPs’ toxicity is to use antioxidants with these MNPs. Ahamed et al. showed an increase in intracellular ROS when human lung adenocarcinoma A549 cells were incubated with nickel ferrite (NiFe_2_O_4_, <50 nm) nanoparticles at 100 mg/mL for 24 h. Furthermore, the antioxidant level of glutathione (GSH) was depleted. The authors observed a significant reduction in ROS and a restoration of GSH with the co-treatment of cells with nickel ferrite nanoparticles and the antioxidant L-ascorbic acid (AA) [99]. Similarly, MNP conjugates with quercetin were fabricated and showed no toxicity while promoting neurogenesis [100].

The size of MNPs is one important parameter for their toxicity in the body. Wu et al. reported that intravenous injection of MNPs in mice with a size below 5 nm exhibited high toxicity, while MNPs with a size over 5 nm did not show any toxicity [95]. However, Ying et al. reported that MNPs generated an immune response in primary macrophages that was size-dependent, with 30 nm inducing a stronger response than 10 nm. Furthermore, the immune response was reduced by blocking ROS stress [101]. Despite conflicting research, ultrasmall MNPs are usually considered as toxic, and globally, an MNP size between 10 and 100 nm looks to be the best for various biomedical applications. Moreover, concentrations of MNPs (SPIONs with various surface coatings) below 100 mg/mL are usually considered to have low or no cytotoxicity [44]. Furthermore, the biodistribution of MNPs is also an important parameter to consider. The biodistribution of MNPs has been observed to be typically 80–90% in the liver, 5–8% in the spleen, and 1–2% in bone marrow [102,103]. In addition, it was observed that MNPs over 200 nm were captured by the spleen, whereas MNPs with a 10 nm size can be removed by renal clearance [102].

Because MNPs’ toxicity is multifactorial, there has been, until now, no clear rules defined for the fabrication of MNPs without toxicity, and new MNPs types must be tested in vitro and in vivo before use. However, several iron oxide-based MNP formulations, such as Fumiren^®^, Feridex^®^, Feraheme^®^, and NanoTherm^®^, have been approved by the FDA and, therefore, are considered safe, having shown no toxicity [91].

#### 2.3.3. Diffusive Properties

Hydrogels are well known as drug delivery systems. This application is based on the control of solute diffusion through the hydrogel matrix. Since solutes, gel polymers, and solvents are the factors that mainly influence the diffusion process, this process is affected by their mutual interactions. Swelling control leads to drug release governed by diffusion and macromolecular relaxation, which gives rise to zero-order circumstances of release [104]. Among drug delivery systems, diffusion-mediated hydrogel systems may be categorized as either reservoir-type or matrix-type. For the reservoir-type systems, the drug is contained in a reservoir which has an aperture closed by a hydrogel membrane through which the drug diffuses [105]. In contrast, for matrix-type devices, the drug is homogeneously mixed in the polymer and diffuses through the whole device.

#### 2.3.4. Biodegradability

The polymeric chains of hydrogels have some bonds that are labile and become broken in aqueous phase, in the presence of specific enzymes (e.g., metalloproteinases, collagenase, hyaluronidases), or by several internal and external stimuli, which results in their degradation. The level and velocity of hydrogels’ degradation in the process of tissue engineering are of special importance. As hydrogels are a scaffold for the growth of tissues, they, at some point, must be biodegradable [106]. Indeed, cells require space to grow; therefore, ideally, hydrogel degradation must synchronously match cell division during tissue repair. Thus, the hydrogel degradation time affects the engineering of tissue, which requires a certain time to be successful. Furthermore, in drug release studies, the degradation of hydrogels also plays an active role in drug release. This hydrogel degradation can be stimulated or slowed down depending on the surrounding conditions of use. It has been reported that through altering the gel content or by using a laser, the breakdown of the hydrogel might be controlled [107].

#### 2.3.5. Stimuli Sensitivity

Smart hydrogels are hydrogels that have shown physical and chemical changes in response to outside conditions. Such stimuli include endogenous factors pertaining to elements such as metal ions, enzymes, pH levels, and antigens, among others, or exogenous factors such as temperature, irradiation, magnetic fields, electric fields, and other additional influences [108,109]. These smart hydrogels have broad applications in sensing, drug delivery, wound healing, shape memory, and tissue engineering. Furthermore, hydrogels that respond to multi-stimuli have also been developed [84].

### 2.4. Properties and Functionalities of Magnetic Hydrogels

MHs possess several properties and functionalities, such as hydrogel mechanical properties, adsorption behavior, magnetocaloric effect, magnetic resonance imaging (MRI), smart hydrogel response, and biocompatibility [110].

#### 2.4.1. Mechanical Properties

**Figure 11:**
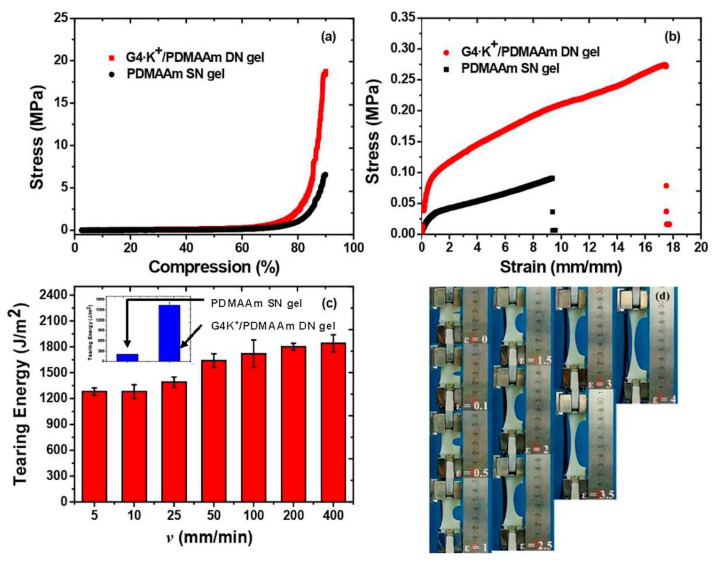
(**a**) The mechanical characteristics of the gels were examined through the compressive stress–strain curve of the PDMAAm SN gel and the G4·K^+^/PDMAAm DN gel. (**b**) The tensile stress–strain behavior of both PDMAAm SN gel and G4·K^+^/PDMAAm DN gel. (**c**) The tearing energies of G4·K^+^/PDMAAm DN gels at various crosshead speeds (the inset figure represents the tearing energies of PDMAAm SN gel and G4·K^+^/PDMAAm DN gel at a tearing crosshead speed of 50 mm/min). (**d**) The crack resistance exhibited by the notched G4·K^+^/PDMAAm DN gel. Reproduced with permission from [111]. Copyright 2018, American Chemical Society.

Mechanical properties represent a collection of essential metrics, defined as the capacity of a material to endure various loads (such as tensile, compressive, torsional, impact, and cyclic loads, among others). Generally, the mechanical characteristics of hydrogels encompass strength, stiffness, toughness, and fatigue resistance, as illustrated in Figure 11, and these mechanical properties significantly influence both the functionality and longevity of hydrogels [111]. Currently, there are four primary approaches to enhance the mechanical properties of hydrogels, as follows:

(1) A ‘‘sacrificial bond’’ is used to reduce the increasing energy in the hydrogels by dissipation, which enhances the mechanical characteristics of the hydrogels. Different non-covalent bonds, such as hydrogen bond self-assembly, complexation, supramolecular recognition, and hydrophobic association, have been used in the design of high-strength hydrogels [111].
(2) The “pulley effect” also helps to lower the internal stress in the crosslinking network and significantly improve the mechanical properties of hydrogels. Thus, topological hydrogel such as polyrotaxane is formed by many cyclic molecules threaded on a single polymer chain terminated by bulky end groups. Such hydrogel has high strength due to O ring-shaped crosslinking points that have high mobility along the polymer chain and equalize the tension in the hydrogel [112].
(3) Reversible non-covalent bonds can also give high strength to hydrogels and a self-healing character by reform after breaking [113].
(4) Hydrogels’ mechanical properties also change when NPs are incorporated into them. Several authors have incorporated nanofillers (e.g., MWCNTs, SWCNTs, GO, metal particles, Laponite, polymeric nanoparticles, clay) in hydrogels to achieve better mechanical properties. These nanocomposite hydrogels are made through the process of radical polymerization of a monomer solution incorporating nanoparticles. Specifically, reagents are adsorbed onto the surface of the nanoparticles, then they start the process of polymerization. It was found that the capping of the polymer ends occurred on the nanoparticles, with the formation of clay/brush particles when clay was used as a nanofiller, and the interactions that occurred were adsorption/desorption and were in no way covalent [114].

#### 2.4.2. Adsorption

**Figure 12:**
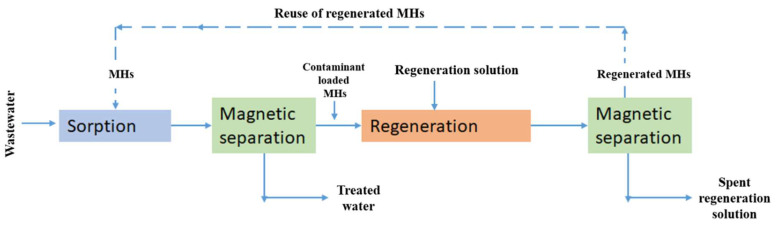
Flowchart of efficient removal of contaminants from wastewater using magnetic hydrogel. First the MHs is added to the wastewater and contaminants adsorbed to the MHs. Then magnetic separation is performed to obtain treated water separated from MHs with contaminants. Then, the MHs is regenerated with a solution to desorb the contaminants from the MHs. The MHs is then re-usable by taking it from the regeneration solution via magnetic separation. Reproduced with permission and with slight changes from [110]. Copyright 2021, The Authors (open access).

Due to their high-water absorption and swelling property, hydrogels have been widely used, for example, in food storage, and as protective agents for agriculture in areas affected by droughts. In addition, due to their high adsorption capacity, hydrogels have great development prospects in the field of wastewater treatment [115]. The procedure of water purification (e.g., removal of heavy metals, pesticides, nitrates, antibiotics, and dyes) based on MH adsorption (e.g., hydrogen bonds, hydrophobic interactions, and electrostatic interactions) is described Figure 12. Thus, fabricated MHs were incorporated into wastewater, and the mixture was subjected to end-over-end shaking to facilitate optimal adsorption of contaminants. Subsequently, the technique of magnetic separation was used to isolate the contaminated hydrogels from the purified water. Moreover, to regenerate the MHs for subsequent utilization, a regeneration solution was applied to eliminate the adsorbed contaminants from the hydrogel surface, followed by magnetic separation to recover the recycled adsorbent materials. Because MHs are cost-effective, repeatedly reusable, and highly efficient adsorbents, they are increasingly used in wastewater treatment processes.

In addition, due to the selectivity of magnetic separation procedures in the recovery of desirable proteins from biological solutions, MH materials have generated lot of enthusiasm in the separation of proteins. Thus, Mahdavinia et al. developed a chitosan/PVA/magnetic laponite RD hydrogel for the adsorption of bovine serum albumin (BSA) as a protein model. Their study showed that the adsorption capacity for BSA increased with the increase in magnetic laponite RD beads in the hydrogel and that the maximum of BSA adsorption was 127.3 mg·g^−1^ at pH 4.5 [116]. Moreover, it is also possible to apply the absorption capacity of MHs to enzyme immobilization [117].

#### 2.4.3. Magnetocaloric Effects

**Figure 13:**
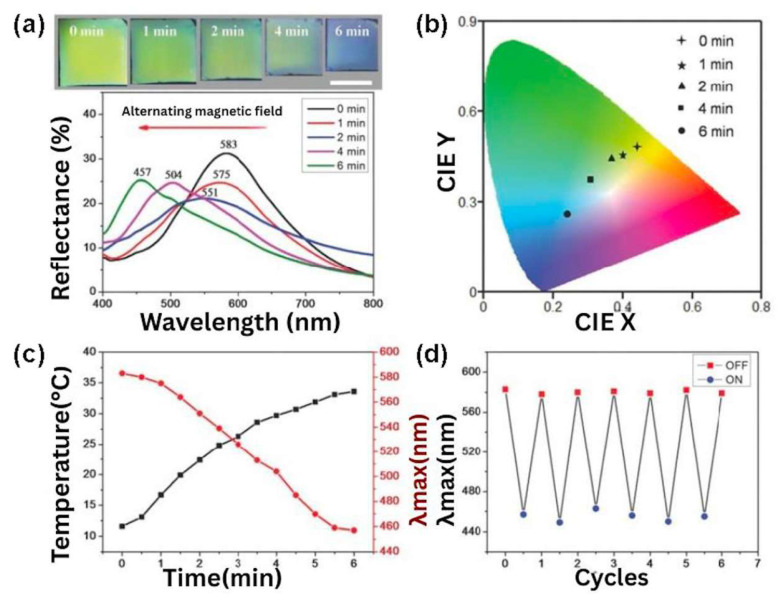
Modification of the physical characteristics of a MH upon exposure to an AMF (1.26 MHz, 1.3 kA m^−1^). (**a**) The photographs depict the chromatic transition of the film over the duration of the AMF stimulation. The diffraction color changes gradually with time from yellow to blue. (scale bar 1 cm). Corresponding reflection spectra captured at various time intervals during the AMF operation. (**b**) The measured peak values of reflection spectra were converted into standard (Commission International de l’Eclairage (CIE)) chromaticity coordinates corresponding to the film at different durations under AMF stimulation. (**c**) Dynamic plots showing the temperature (square symbol) and λ_max_ (circular symbol) of the film under the AMF. (**d**) Diffraction wavelengths recorded over 6 cycles with the AMF applied for 6 min (AMF ON). For replication, this process was reiterated, with the film being cooled back to its initial temperature of approximately 11 °C after the magnetic heating was ceased (AMF OFF). Reproduced with permission from [122]. Copyright 2017, WILEY-VCH Verlag GmbH & co.

The magnetocaloric effect (MCE) is the trend of a magnetic material to become hot or cool when placed in an AMF [118,119]. This phenomenon has important applications in medicine, especially in tumor ablation and drug delivery systems. Thus, in nanomedicine, many studies have used SPIONs for tumor ablation by locally increasing the heat after irradiation with near-infrared light (NIR), electromagnetic, or radio frequency waves. Moreover, these NPs can be functionalized to enhance their selectivity toward cancerous cells and for multitasking such as cell separation and imaging [120]. However, there are several issues related to the use of SPIONs for hyperthermal therapy, since they showed a high clearance rate in vivo, low time resolution if used with AFM, and may require multiple injections. Moreover, when using MHs, the hydrogel matrix, with its internal three-dimensional interconnected structure with a high water content ratio, can be loaded with a drug, and the magnetocaloric effect can be adjusted to maintain and regulate the release of one or several drugs [14]. Thus, MHs can combine hyperthermia therapy (with an effective therapeutic heat temperature usually around 42 °C), due to its magnetic component, with drug delivery therapy due to its hydrogel component. Advantageously, this combined strategy using nanoMHs allows for reaching tumors located deep in the body like liver cancer or glioma. Incidentally, several in vitro cell studies have also identified that mild thermal stimulation might enhance osteochondral regeneration [121]. Another application of the magnetocaloric effect is in stimuli-responsive chromic materials in the field of display. Thus, Wang et al. developed magnetochromic photonic hydrogels that display colorimetric responses (Figure 13) when placed in an AMF [122]. Thus, under a magnetic field, Fe_3_O_4_@SiO_2_ colloids formed 1D magnetic chains in a thermosensitive copolymer of N-isopropylacrylamide (PNIPAM)/polyethylene diacrylate (PEGDA). This photonic structure was fixed with UV irradiation. Then, when placed in an AMF, the magnetocaloric effect of the aggregated magnetic chains caused a change in the hydrophilic–hydrophobic balance of the hydrogel, leading to a decrease in the inter-particle distance of the magnetic chains in the MHs, which induced a shift in the diffraction wavelength toward the blue side of the spectrum (Figure 13).

#### 2.4.4. Swelling Behavior

Hydrogels have a swelling property in aqueous environments. This swelling is naturally occurring, compressible, and dissipative, and hydrogels can hold large amounts of aqueous fluids within their interconnected structure without chemical changes. This swelling is mainly attributed to the rather low crosslinking density of the hydrogel network and the significant number of hydrophilic functional groups (including amino, hydroxyl, and carboxyl) present on macromolecular scaffolds. Thus, hydrogel swelling increases when the hydrogel crosslinking decreases, and this swelling can lead to categorizing certain hydrogels as high-swelling hydrogels (HSHs) with a swelling ratio of >150% [123]. These HSHs can be formed from natural polymers and synthetic polymers or from a mixture of both. The swelling capacity of a hydrogel is an important property in tissue engineering and drug delivery systems. However, constant swelling or low swelling may be required, especially during hydrogel degradation, when the polymer chains become less entangled. To this end, non-swelling hydrogel (NHSs) with a swelling ratio of 0–150% have been fabricated and are a specialized form of hydrogel that undergoes very little swelling in water [124]. Some common strategies, among others, for the fabrication of NSHs include an increase in the crosslinking density of the hydrogel, which limits the intake of water molecules and improves the mechanical properties of the hydrogel, the modulation of the polymer/water ratio with the introduction of hydrophobic segments in the hydrophilic polymer chains, and the use of thermosensitive polymers with a low critical solution temperature (LCST) that will shrink above LCST, impairing swelling [123,124].

#### 2.4.5. Intelligent Response

**Figure 14:**
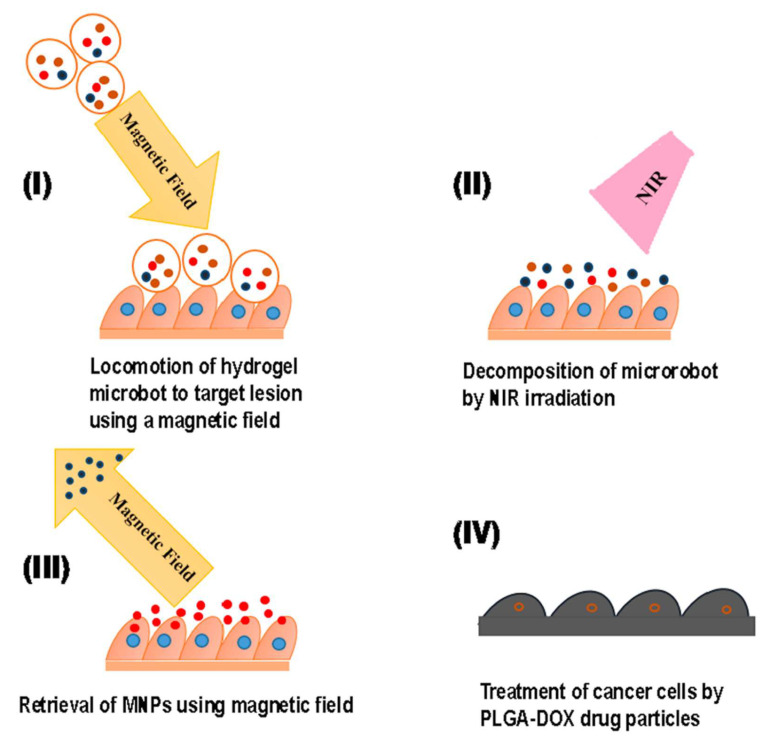
Schematic of the treatment process of cancer cells with hydrogel microrobot. (**I**) First, the hydrogel microrobot carrying MNPs and PLGA–DOX drug particles navigated under magnetic field to the targeted position. (**II**) Second, under NIR irradiation the MNPs release heat that degrades the gelatin/PVA hydrogel of the microrobot releasing the cargo PLGA-DOX drug particles. (**III**) Then the MNPs are removed from the body with the magnetic field, (**IV**) while the remaining PLGA-DOX particles degrade slowly treating the cancer cells. Modified with permission from [128]. Copyright 2019 Elsevier B.V.

As noted previously in Section 1 (classification of hydrogels), dynamic hydrogels respond to a variety of external stimuli (e.g., pressure, strain, temperature, light, pH, ions, magnetic field). Such hydrogels have found applications in tissue engineering, soft actuation, and drug delivery [125]. Among various external stimuli applicable to stimuli-responsive materials, the magnetic field exhibits advantages such as immediacy, non-contact control, and compatibility with electronic devices. As a result, the development of new MHs has gained considerable momentum in recent years, as shown Figure 14 [126]. Over the past several decades, tissue engineering techniques have been used extensively in the repair and regeneration of diverse tissues, including retinas, ligaments, adipose tissue, and vascular structures. On the one hand, magnetic hydrogels possess the capability for directional movement and can be directed into specific tissue-like microstructures (e.g., multilayers, aggregates) through the application of magnetic fields to facilitate tissue reconstruction [78]. Li et al. developed a new hydrogel actuator that was designed with the ability to release anticancer drugs through both NIR and electromagnetic actuation (EMA) and for the retrieval of MNPs from a hydrogel microrobot. For movement in the three dimensions, the EMA system uses Helmholtz coils and Maxwell coils. The Helmholtz coil pair creates a constant magnetic field density, while the Maxwell coil creates graded flux magnetic density. While the positioning of the microrobot in the required direction can be made by the Helmholtz coils, the actual movement of the microrobot in the desired direction can be made along the Maxwell coils. Thus, through the EMA magnetic field, the microrobot navigated to the targeted position at 600 μm/s. Following NIR irradiation, the hydrogel matrix underwent degradation, resulting in the retention of drug-loaded particles and magnetic nanoparticles (MNPs) at the tumor site. Ultimately, after 6 h of treatment facilitated by EMA, the disintegrated MNPs were segregated from the targeted site, while the residual anticancer drug-loaded particles persisted in releasing the drug to exert therapeutic effects. This hydrogel actuator addresses the limitations associated with MNPs’ potential accumulation and toxicity, by eliminating MNPs while simultaneously preserving the benefits conferred by electromagnetic drive, including targeted delivery and drug release [127,128].

## 3. Biomedical Applications of MHs

**Figure 15:**
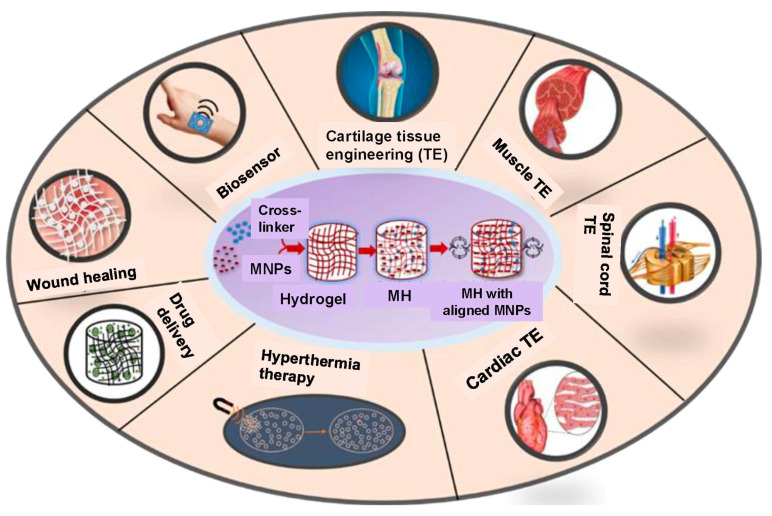
Schematic of biomedical application of MHs.

The biomedical applications of polymeric materials can be performed with improved effectiveness with the incorporation of MNPs [129]. Indeed, the hydrogel part of MHs can be used for the diffusion of small molecules in and out of the gel matrix, while the MNPs can react to a magnetic field passing through biological tissues and organs [130]. This fusion leads to a versatile material for several applications, including the delivery of drugs, hyperthermia treatment, imaging, wound healing, biosensing, and tissue engineering (Figure 15). In the following sections, we detail the different applications of MHs.

### 3.1. MHs in Drug Delivery

**Figure 16:**
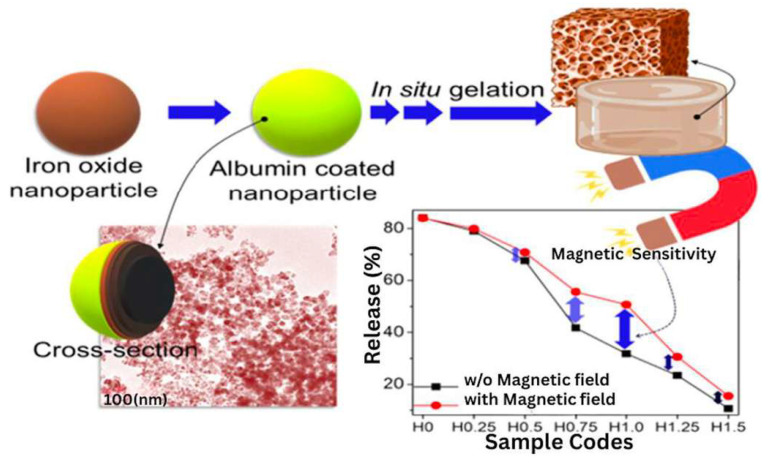
Schematic illustration showing the functionalization of iron oxide nanoparticles with albumin for controlled drug release by magnetic diffusion from the hydrogel layer with and without an applied magnetic field. Reproduced with permission from [43]. Copyright 2024, American Chemical Society.

Hydrogels are well known for their efficiency as drug carriers and drug delivery systems [131,132]. In particular, a lot of developments for drug delivery are made in stimuli-responsive hydrogels [133]. However, due to the nature of these stimuli (e.g., pH, light, ultrasound, near red radiation), the application of the hydrogel becomes specific to a biological tissue (Figure 16). Interestingly, this limitation of tissue-specific stimuli can be overcome by using hydrogels loaded with nanoparticles, which enhances the responsiveness of these hydrogels [134]. In particular, hydrogels containing magnetic particles have been used in various applications, and numerous reports have shown that drug delivery can be accomplished with MHs. For example, Ganguly et al. enumerated works based on SPIONs-arrested hydrogel matrices as some of the promising materials in smart soft biomaterials. They reported the fabrication of superparamagnetic amine-functionalized maghemite nanoparticles (SPIONs) and their encapsulation in a semi-IPN hydrogel of poly (acrylic acid-co-hydroxyethyl methacrylate) for drug release applications. They showed that SPIONs acted as a mechanical reinforcing and rheological modifying agent of the hydrogels. Furthermore, they observed that two factors controlled the drug release behavior of the hydrogel, including changes in pH and static magnetic fields. In particular, at a low SPIONs concentration, drug release was mainly dependent on pH, while at a high SPIONs concentration (~1%), drug release was mainly dependent on the magnetic field [43]. In another study, Lin et al. described a method to enhance the functionality of cellulose hydrogels for remote drug release applications. They fabricated magnetic β-cyclodextrin (β-CD)/cellulose hydrogel beads with a one-step procedure. These hydrogels showed fast swelling and deswelling behavior and allowed for modulated drug delivery in an external magnetic field (EMF). Furthermore, the presence of grafted β-CD enhanced drug loading, while the drug release dose and rate could be adjusted by turning the EMF on and off and by varying the Fe_3_O_4_ nanoparticle concentration. In addition, cytotoxicity tests confirmed the non-toxicity of the material [135]. Furthermore, Ribeiro et al. fabricated an injectable xanthan gum hydrogel with Fe_3_O_4_-based MNPs. This composite hydrogel was designed to deliver drugs via hyperthermia and be visualized with MRI. The hydrogel showed a high loading efficiency with hydrophilic drugs and MNPs. In addition, when an MH containing 10% MNPs was put under an AMF, the temperature increased to 60 °C, showing good hyperthermia potential. Furthermore, the hydrogel shortened the water proton relaxation time, suggesting its potential for application as a T2-MRI contrast agent at a clinical field strength of 3 Tesla. Finally, the drug release was enhanced by three times under a magnetic field compared to no magnetic activation [136]. Moreover, Chen et al. fabricated a PVA hydrogel encapsulating Fe_3_O_4_ MNPs. When this hydrogel was subjected to on–off intervals of magnetic field, the pore size of the hydrogel decreased under the activation of the magnetic field and, therefore, less drug was released. The study showed that when the MH was injected in nude mice bearing hepatoma, the gathering of the MNPs around the tumor, combined with the effect of an extremely low-frequency altering electric magnetic field (ELFF), significantly inhibited tumor cell proliferation compared to other mice groups and prolonged their survival time [137]. Another feature of drug-loaded MHs is their ability to achieve drug-controlled sites in living organisms after their introduction into an external magnetic field [138]. Indeed, when a magnetic field is applied to media containing MHs, the spins rotate and reorient themselves. In addition, the hysteresis loop of the system changes and, thus, it is possible to remotely control the targeted delivery systems [139].

### 3.2. MHs in Hyperthermia

**Figure 17:**
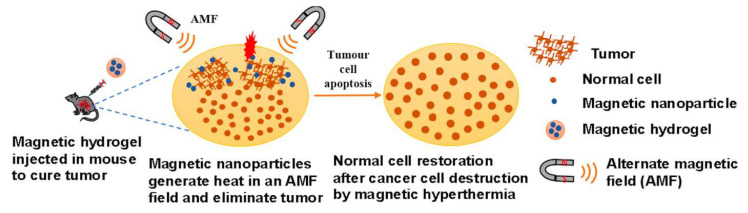
Explanatory diagram of MNP-mediated MHT for selective tumor cells ablation.

Over the past decades, cancer has been the number one reason for the incidence of death, and the death toll is increasing daily. Cancer is usually treated using surgery and other forms of cancer therapy such as radiotherapy and chemotherapy. However, a less invasive therapy is hyperthermia, which uses the local rise in temperature to destroy cancerous cells with a minimal impact on the surrounding normal cells [140]. In addition, it is possible to combine hyperthermia and drug delivery to obtain synergistic effects [141]. Recurrent problems in bone tumor therapy are tumor recurrence and metastases and tumor localization, which can be rather deep in the body. Recently, Hu et al. fabricated an injectable doxorubicin (DOX)-loaded magnetic alginate hydrogel (DOX@MAH) for bone tumor applications. This hydrogel showed a high drug loading capacity and effective controlled release of DOX. Applied in vivo in tumor-bearing mice, the injection of DOX@MAH showed efficient antitumor activity under AMF with the combination of hyperthermia and chemical therapy [17]. Similarly, to tackle melanoma and metastases with hyperthermia and drug therapy, Sumitha et al. developed magnetic patches of a chitosan-TEMPO oxidized nanocellulose film loaded with SPIONs and DOX. These patches, containing uniform SPIONs, were characterized and showed biodegradability, cytocompatibility, and a higher hydrogel swelling ratio at tumor pH than at physiological pH. Moreover, the patches were superparamagnetic, with a saturation magnetization of 23.3 emu/g. A drug release study with an encapsulated dye used as a model drug showed that under an EMF of 50 mT, more than 12% of the total dye loaded amount was released in 1 h, while without EMF, a similar release level would have taken more than 24 h. The patches were also tested for hyperthermia activity, and it was observed that the patches under EMF were able to raise the temperature above 42 °C in seven minutes. Furthermore, with 50 μg/mL DOX loaded patches, the viability of B16F10 murine melanoma cells was reduced by 79.55% in five days [142]. Mild hyperthermia usually uses a thermal window of 41–46 °C. However, tumor cells under heat stress may activate the autophagy process as a mechanism of defense and restoration, which may result in some thermal resistance. To overcome this and boost mild magnetic hyperthermia therapy (MHT), Wang et al. chose to fabricate a nitric oxide (NO)-releasing MH, because NO impairs the autophagy process. Thus, they developed an injectable hydrogel of thermosensitive poly(ethylene glycol)-polypeptide copolymers modified with NO groups on their side chains (Figure 17). They also synthesized ferrimagnetic Zn_0.5_Fe_2.5_O_4_ MNPs (cubes, 70 nm), which are very popular for their ability to deliver high magnetic–thermal conversion, and incorporated them directly within the hydrogel to form MNPs@NO-Gel. The MNPs@NO-Gel was tested in vivo in mice bearing CT-26 tumor cells (~100 mm^3^) who received three mild MHT treatments (20 min at 42.5°, H 17.6 kA/m, f 282 kHz) at days 0, 2, and 4 after the injection of the MNPs@NO-Gel. At day 14, the mice were euthanized and their tumors were analyzed. The results showed that in the group of mice treated by the MNPs@NO-Gel with MHT three times, the tumors were eliminated, and the survival of mice was 100% with no recurrence. Furthermore, the study globally showed that several sessions of MHT were possible after only one injection of gel due to the homogeneous distribution and strong adhesion of MNPs in the gel phase. Moreover, nitric oxide (NO) was continuously released from NO-Gel, and this release was boosted by MHT. The degradation of MNPs@NO-Gel in vivo was slow, with 30% of MNPs@NO-Gel remaining after 35 days, whereas the dispersion of the released MNPs was concentrated in the spleen. Due to the release of NO controlling the autophagy activity, there was suppression of the formation of autophagosomes and lysosomes during MHT, which boosted the efficiency of MHT [143]. In a recent study, Barra et al. assessed the performances of chitosan-based magnetic composite films in terms of film thickness, MNP content, and heating efficiency. In particular, spatiotemporal heating was evaluated with a thermal camera, and heat maps were generated. Furthermore, the polymeric films were used in MHT treatment under AMF (663 kHz, 12.8 kA/m) with MNT-1 human melanoma cells. The polymeric films of chitosan/glycerol/MNPs obtained with a casting method were very thin, with thicknesses ranging from 34 μm to 93 μm. The thermal efficiency of the films increased significantly when exposed to an AMF with a maximum of 82 °C recorded within 270 s. The temperature increased with an increase in film thickness and an increase in MNP content. Moreover, in the MHT treatment experiment with a 78 μm film thickness (labeled 2.25 M-0.75G-T), the temperature reached 42 °C within 300 s, and after this temperature was maintained for 10 min, the viability of MNT-1 cells decreased drastically to 8% [144].

### 3.3. MHs in MRI

**Figure 18:**
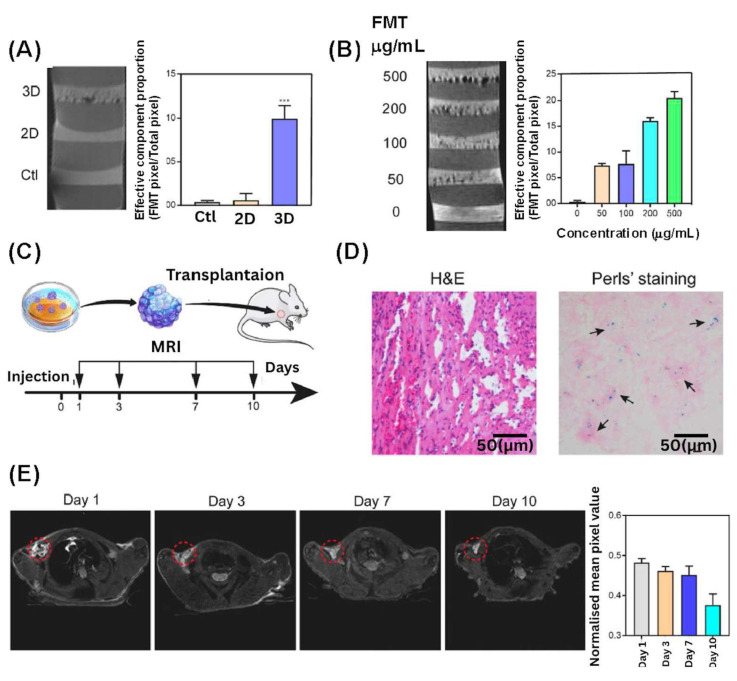
Characterization of ferumoxytol-labeled spheroids was conducted utilizing both in vitro and in vivo magnetic resonance imaging techniques. This figure illustrates (**A**) longitudinal T2 MRI sections of agarose infused with cells cultured on distinct hydrogels, accompanied by a relative quantitative analysis. (**B**) T2 MR images of spheroids subjected to varying concentrations of ferumoxytol at 0, 50, 100, 200, and 500 μg mL^−1^ are presented alongside a quantitative analysis of contrast enhancement. (**C**) In the context of this study, protocol designed for MRI monitoring post-transplantation in nude BALB/c mice. (**D**) Hematoxylin and eosin staining and Perls staining. (**E**) Displays of axial T2 MR images of mice at 1, 3, 7, and 10 days after the transplantation of labeled hUC-MSC spheroids, along with a relative quantitative analysis. Reproduced with permission from [146]. Copyright 2022, The Authors (open access).

Molecular imaging in general and non-invasive imaging in particular are advantageous, and may come with the best feedback for clinical diagnosis. With the advantages of safety, functional sequences, good contrast, and penetration depth, magnetic resonance imaging (MRI) is one of the most powerful detection methods in contemporary clinical diagnosis. However, in practical situations, the relaxation times of different tissues or tumors are of the same magnitude, which makes diagnosis very complex. Hence, contrast agents began to be developed to enhance the signal contrast to improve the image resolution. Due to their biocompatible and superparamagnetic properties, Fe_3_O_4_-based superparamagnetic contrast agents are used in cancer detection, monitoring of drug delivery, and labeling of steel stents. Thus, research on contrast agents is a dynamic field of research [145]. Yan et al. studied the labeling of MSCs with SPIONs as a method of MRI tracking of transplanted cells in tissue repair research and clinical trials (Figure 18). Previously, the authors reported that MSC labeling with clinically safe SPIONs (ferumoxytol) was possible by using only transfection reagents or a magnetic field, which greatly limited their application in clinic. To overcome this problem, this new study showed that the magnetic labeling of MSC spheroids using ferumoxytol was performed by encapsulating the ferumoxytol into the spheroids and by its binding with the ECM. The results showed strong MRI T2 signals of the labeled MSC spheroids and a higher biosafety for the MSCs, demonstrating the potential application of this method in post-transplantation MRI in the clinic [146]. Recently, Mistral et al. studied the effects of a polymeric coating of chitosan (CS) on SPIONs for use as MRI contrast agent. They used CS with the same degree of polymerization DPw = 450) and with different degrees of acetylation (1%, 14%, 34%). The synthesized SPIONs were spherical with a size of 5–10 nm and a highly crystalline magnetite phase. The CS coating improved the biological properties of the SPIONs, but the thickness of the coating decreased the magnetic property. The authors concluded that the best CS coating of SPIONs was for a thin shell (20%) with a low degree of acetylation (1%) [147].

### 3.4. MHs in Wound Healing

**Figure 19:**
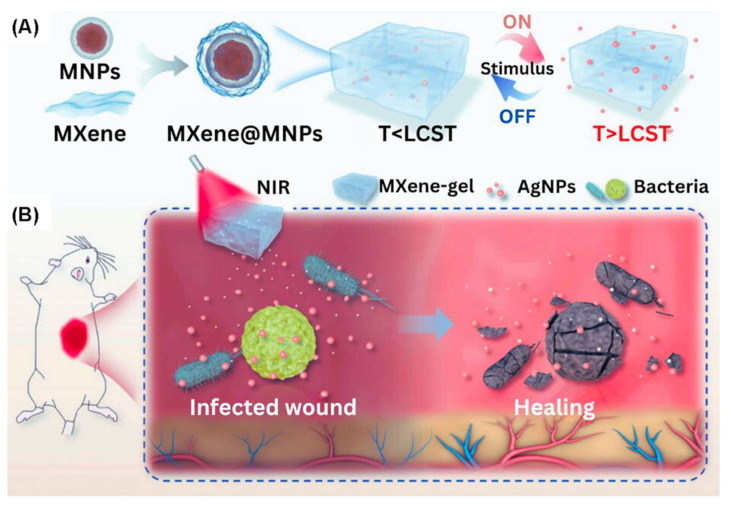
Illustration of syntheses and uses of stimuli-responsive MXene-based hydrogel system for bio-, thermo-, and pH-responsive applications. (**A**) The synthesis mechanism of the MXene-based hydrogel system and the mechanism of drug release in this system. (**B**) Deep chronic infected wound model treated with non-invasive red-light-sensitive AgNPs/MXene hydrogel system. Reproduced with permission from [153]. Copyright 2021, Wiley-VCH GmbH.

The process of wound healing naturally occurs after injury and is usually divided into four stages, which overlap each other. These involve hemostasis, inflammation, and cell proliferation, as well as tissue remodeling. However, if the trauma is too significant, for example, in cases of excessive inflammation, burns, accidents that lead to the loss of a large area of skin tissue, infection, and diabetes, then the process of wound healing will be affected. Wound dressings can provide a barrier by simply covering the wound, which prevents other irritating materials from entering into contact with the wound area, and they can, in addition, provide a morphogenic pattern where skin cells arrange themselves in a particular order. An ideal skin wound dressing should meet the following requirements: (1) the dressing cannot be toxic or cause inflammation; (2) the dressing should provide good moisture retention and allow for some absorption of wound exudate; (3) the dressing should have a sufficient physical and mechanical strength to ensure its integrity and avoid the intrusion of external bacteria caused by materials’ breakage; and (4) the dressing should have an appropriate surface microstructure and biochemical properties to promote cell adhesion, proliferation, and differentiation [148]. Various wound dressing formats include gauze, semipermeable membranes, semipermeable foams, hydrocolloids, and hydrogels [149]. Among them, hydrogels have emerged as the most promising candidates for wound dressings because of the following three characteristics: good hydrophilicity, excellent biocompatibility, and a 3D porous structure mimicking the ECM [150]. Recent developments have occurred with the fabrication of hybrid hydrogels and smart hydrogels (like MHs), with use in wound healing. These hydrogels consist of natural and/or synthetic polymers which can achieve some functions enhanced by the addition of active nanofillers [151]. Different approaches for the synthesis of antibacterial hydrogels have been reported by the incorporation of antibacterial agents (e.g., antibiotic, antibacterial drugs, and metal nanoparticles) in the hydrogel network through physical chemical crosslinking [152]. Thus, Yang et al. incorporated a thin shell of SiO_2_ onto Fe_3_O_4_ nanoparticles to obtain Fe_3_O_4_ @SiO_2_ MNPs. These MNPs were then functionalized with a thin layer of MXene (Ti_3_C_2_) to produce (MNPs@MXene) with a high photothermal conversion efficiency (PTCOE). MNPs@MXene was incorporated into a thermo-sensitive PNIPAM/alginate composite hydrogel (Figure 19). Moreover, to impart antibacterial characteristics to the hydrogel, AgNPs were loaded into the hydrogel to form PNIPAM/alginate/MNPs@MXene/AgNPs. Thus, MNPs were heated up on exposure to NIR light or through magnetic stimulation from an AMF, and the hydrogel swelled, inducing controlled release of the encapsulated AgNPs, which provides a proper antibacterial response [153].

**Figure 20:**
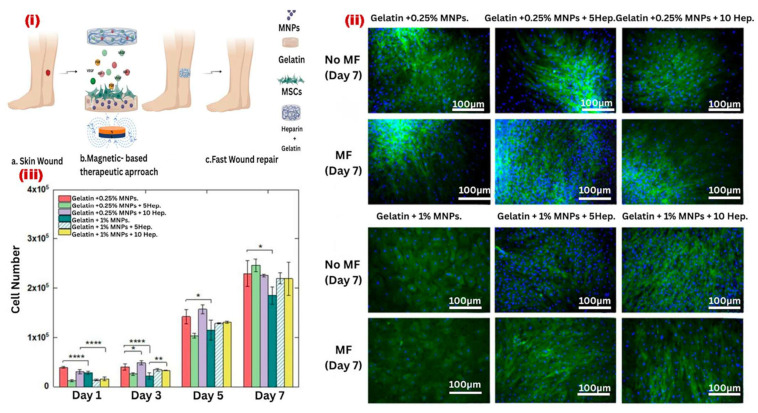
(**i**) Schematic of heparinized magnetic hydrogels serves as a promising approach to promote angiogenesis in different types of tissue damage. (**ii**) Fluorescence microscopy images of MSCs stained with DAPI/FITC–phalloidin in non-heparinized and heparinized magnetic hydrogels on the seventh day post-seeding. Green fluorescence represents actin filaments, while blue fluorescence indicates cell nuclei. (**iii**) MSC proliferation on non-heparinized and heparinized hydrogels containing 0.25% and 1% MNPs after exposure to a 0.08 T magnetic field (MF) for 24 h. Each asterisk (*) denotes a statistically significant difference (*p* < 0.001). We assume that more there are * more the significance was. Reproduced with permission from [154]. Copyright 2024, American Chemical Society.

Pires et al., [154], investigated the regulation of angiogenesis as a potential strategy for therapeutic applications in cancer treatment and wound healing (Figure 20). In this study, MSCs were cultured on magnetically responsive gelatin scaffolds, with and without heparin functionalization, and exposed to a static 0.08 T magnetic field (MF) to modulate their cellular behavior. For the first time, the impact of heparin and MNPs’ distribution within gelatin scaffolds on hydrogel mechanical properties, MSC morphology, proliferation, and secretome profiling was analyzed. The results revealed that incorporating MNPs and heparin influenced hydrogel swelling behavior and MSC proliferation rates. Additionally, MF provided a topographical stimulus, aligning MSCs and upregulating genes and proteins associated with angiogenesis. Interestingly, higher heparin concentrations (10 μg/cm^3^) restricted angiogenic factor diffusion into the culture medium. Ultimately, acellular heparinized hydrogels effectively retained angiogenic growth factors secreted by magnetically stimulated MSCs, leading to superior wound contraction (55.8% ± 0.4%) and cell migration (49.4% ± 0.4%) compared to non-heparinized hydrogels (35.2% ± 0.7% and 37.8% ± 0.7%, respectively). These findings suggest that heparinized MHs hold significant potential for promoting angiogenesis and enhancing tissue regeneration in bone defects, skin wounds, and cardiovascular diseases [154].

**Figure 21:**
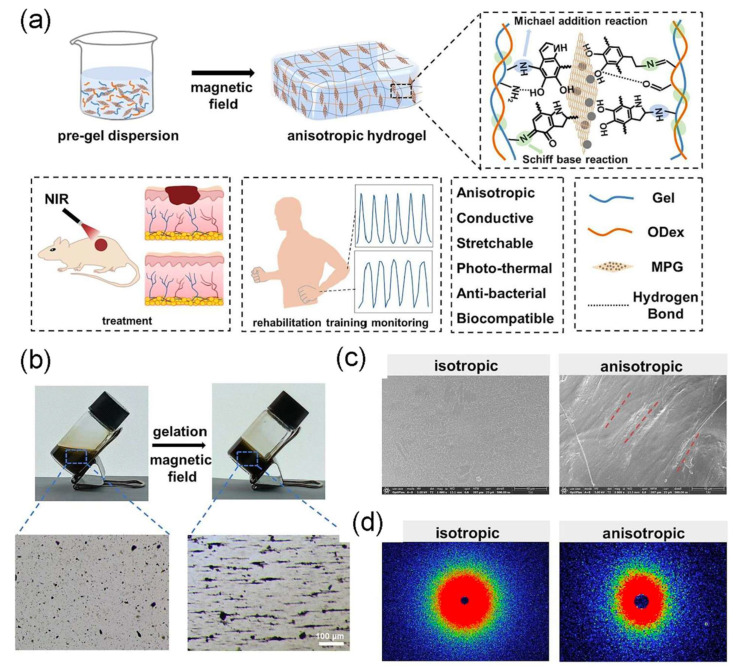
Formation of the anisotropic GOH-MPG conductive hydrogels: (**a**) Schematic illustration showing the preparation process of the anisotropic GOH-MPG conductive hydrogel and its application in the medical field. (**b**) Optical images displaying the formation of the anisotropic GOH-MPG hydrogel and the distribution of MPG nanosheets. (**c**) SEM images comparing isotropic and anisotropic hydrogels (GOH-MPG1.0), with alignment indicated along the red line. (**d**) Two-dimensional SAXS patterns of both isotropic and anisotropic hydrogels. Reproduced with permission from [155]. Copyright 2023, Elsevier B.V.

Anisotropic Gel-ODex-MPG hydrogels, developed by Li X. et al. (Figure 21), integrating magnetoelectric nanosheets (Fe_3_O_4_-PDA-rGO) into gelatin-oxidized dextran under a magnetic field, exhibit multifunctionality for wound healing. These hydrogels possess anisotropic mechanical and conductive properties, along with strong photothermal antibacterial activity under near-infrared (808 nm) light. They not only accelerate healing of infected wounds, but also monitor human motion, enabling real-time wound status assessment and rehabilitation training. Inspired by natural soft tissue structures, this study marks the first combination of anisotropic hydrogels with wearable sensors, offering a novel approach for simultaneous treatment and recovery monitoring in wound management and personalized healthcare applications [155].

### 3.5. MHs in Bio-Sensing

**Figure 22:**
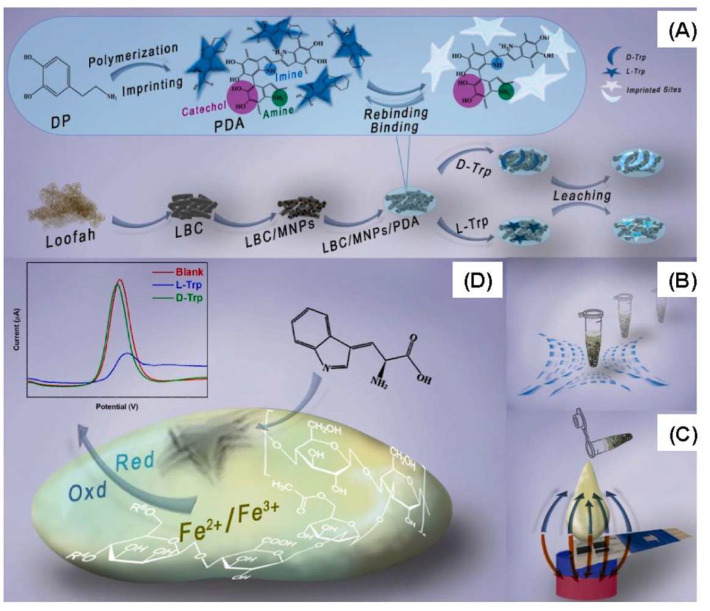
Schematic representation delineating the procedural steps for the preparation of LBC-Fe_3_O_4_/-MIPDA (**A**), the impregnation of LBC-Fe_3_O_4_/-MIPDA within XG and the associated sample pre-treatment (**B**), the drop casting of the formulated mixture onto a magnetite SPE (**C**), and the functional mechanism underlying magnetic redox-responsive detection (**D**). Reproduced with permission from [162]. Copyright 2024, Elsevier B.V.

The creation of MHs has gradually accelerated because of their versatility in the field of sensing [156]. A sensor is composed of a recognition part which detects the targeted analyte and of a transducer part which converts the detection event into a measurable signal [157,158,159]. Among sensors, biosensors are highly sensitive, rapid, and accurate diagnostic tools for detecting biomarkers and pathogens with real-time monitoring, enhancing the diagnosis of diseases [160]. Thus, Kim et al. fabricated a PGA–chitosan (CS) hydrogel nanoparticles for encapsulating both glucose oxidase (GOx) and MNPs in the hydrogel matrix for the detection of glucose. GOx and MNPs were mixed with PGA solution, which was then added dropwise into the CS solution to form rapidly gelling hydrogel nanoparticles. In presence of glucose, the GOx converts the glucose into gluconolactone, producing hydrogen peroxide (H_2_O_2_), which activates the peroxidase-like activity of MNPs to oxidize the chromogenic substrate 2,2-azino-bis (3-ethylbenzothiazoline-6-sulphonic acid) diammonium salt (ABTS) into a green-colored product. Using this approach, the glucose was found to be measurable in the linear range from 5 to 100 μM with an LOD of 3 M, allowing for diagnosis of hyperglycemia in human blood. Furthermore, the sensor showed high stability and magnetic reusability [161]. In another study, Zhang et al. fabricated a wireless and passive flexible strain sensor based on gelatin methacrylate (GelMA)/Fe_3_O_4_ as an MH. This sensor has the advantages of real-time and continuous monitoring without the disadvantages of wires and power supplies. The sensor is compliant with ultrasoft mechanical behavior (Young’s modulus 1.2 kPa), possesses robust magnetic attributes (12.74 emu/g), good biocompatibility, long-term stability (>20 days), can function for long hours in saline solution, and is sensitive enough to detect small strains down to 50 μm. The study comprises a model for the appropriate positioning of the sensor and its magnetic permeability and sensitivity. Moreover, cardiomyocytes were seeded on the strain sensor, and the effects of the cell density, contractibility, and drugs were evaluated. This research showed the feasibility of using a magnetic-sensing approach for biomechanical measurements and highlights the developments of wireless and fully passive implantable devices [19]. Recently, Hosseini et al. fabricated a magnetic molecularly imprinted polymer (MIP) sensor (Figure 22) for the enantiomeric detection of the essential amino acid L-Tryptophan (L-Trp). The sensor was developed utilizing oriented biochar derived from Loofah (LBC), Fe_3_O_4_, and molecularly imprinted polydopamine (MIPDA) embedded within xanthan gum (XG) hydrogel. The template (L-Trp) was removed by immersion in a solution of methanol/acetic acid (ratio 9:1) under sonication. To form the sensor, the prepared hydrogel (XG-LBC-Fe_3_O_4_/MIPDA) was then drop-cast on a screen-printed electrode (SPE). The synthesis of these components was validated through comprehensive physicochemical and electrochemical analyses. Several operational parameters, including pH, response time, sample loading volume, and the quantity of active materials to be incorporated were meticulously optimized. The SPE-XG-LBC-Fe_3_O_4_/MIPDA sensor showed a linear detection range of L-Trp from 1 to 6 μM and from 10 to 60 μM, with an LOD of 0.44 μM. Moreover, the electrochemical sensor exhibited good reproducibility and selectivity. In addition, the detection of L-Trp in milk, blood, and urine samples was good, with a relative standard deviation (RSD) ranging from 97% to 106%. The strategy provided in the development of this sensor is promising, convenience, and effective. It uses xanthan hydrogel for enhancing mass transfer and adhesion strength, Fe_3_O_4_-supported biochar for orientating magnetic fields and accelerating mass transfer and sensitivity, and polydopamine MIP for selectivity. This approach makes it easy to assess the L-Trp levels on-site, which is quite useful in health assessment and early diagnosis associated with L-Trp [162].

## 4. Other Applications of MHs in Tissue Engineering

### 4.1. Applications of MHs in Neural Tissue Engineering

Neural tissue is responsible for the conduction of nerve impulse. Neurons are the main component of the neural tissue and are located in the central nervous system (CNS) and in the spinal cord. They are excitable cells sending and receiving signals through action potentials. They have a cell body (or soma) and elongated projections such as axons, which end at presynaptic terminals containing boutons, and dendrites, which form variable branching. There are different types of neurons, which are often classified into sensory neurons (carry information toward the CNS or spinal cord), interneurons (relay information within the CNS or spinal cord), and motor neurons (send information out of the CNS or spinal cord) [163]. Neural tissue is among the most tender tissues in the whole human body, with an elastic modulus well below 1 kPa [164].

**Figure 23:**
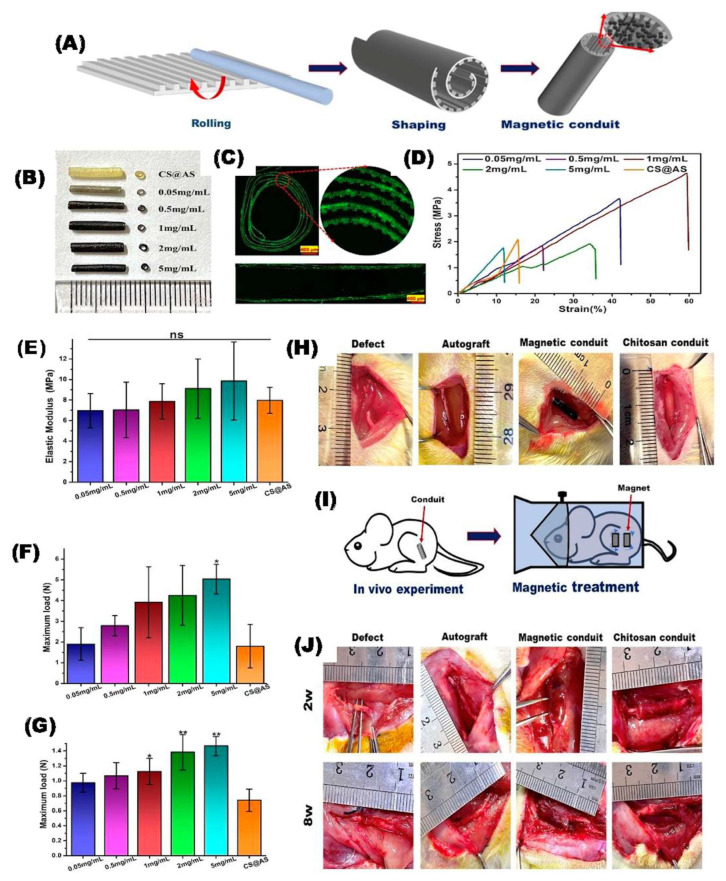
Synthesis and characterization of magnetically responsive anisotropic topological tubes for nerve guidance. (**A**) Schematic representation of the fabrication methodology. (**B**) Macroscopic assessment of the conduits with various degrees of dopamine-modified Fe_3_O_4_ (DFe_3_O_4_) incorporation. (**C**) A fluorescence analysis of both transversal and longitudinal sections of the conduit containing 2 mg/mL DFe_3_O_4_. (**D**–**G**) The stress–strain relationship, elastic modulus, peak loading force, and suture strength of the magnetic conduit with different concentrations of DFe_3_O_4_ (0.05, 0.5, 1, 2, 5 mg/mL and control (from violet to orange)), (*n* = 6), ns (non significant), * *p* < 0.05, ** *p* < 0.01 compared to control group. (**H**) Photos showing the sciatic nerve injury (1 cm nerve gap) in a rat model, and the position of the nerve conduit bridging the defect. (**I**) A schematic representation of the magnetic interference processing for rat treatment. (**J**) Macroscopic images of the surgical site following 2 weeks and 8 weeks post-surgery. Reproduced with permission from [170]. Copyright 2024, Elsevier B.V.

Neural injuries are still difficult to treat. However, one approach in neural tissue engineering is the development of nerve guidance conduits using biomaterials, which provide a suitable environment for neuron survival and axon extension [165]. Among these biomaterials, the use of hydrogels is particularly adapted due to their low Young’s modulus. Moreover, the use of smart materials such as MHs is also well developed. Thus, Antman-Passig and Shefi fabricated an injectable anisotropic MH of collagen for neural tissue engineering. They dispersed magnetite and maghemite MNPs within the collagen solution and used a weak magnetic field (25.5 mT) during the gelling process to aggregate the MNPs into string-like clusters aligned with the orientation of the magnetic field within the hydrogels. These physical cues promoted the elongation of collagen fibrils during gelation and the alignment of neurites from encapsulated primary neurons by contact guidance. Furthermore, when PC12 cells (pre-neural cells) were encapsulated into the MH collagen gel under a magnetic field and their differentiation was stimulated with nerve growth factor (NGF), the neurites’ branching tree was significantly oriented [40]. In addition, Tay et al. examined the effects of magnetomechanical modulation on primary dorsal root ganglion (DRG) neurons using magnetic microparticles (MMPs) incorporated in high-molecular-weight hyaluronic acid hydrogels. This MH was meticulously engineered to emulate the extracellular matrix (ECM) of the spinal cord with a storage modulus of 0.14 kPa. The MH promoted the survival and the healthy outgrowth of DRG neurons, enhancing the expression of both excitatory and inhibitory ion channels, as observed under confocal microscopy. In this study, the authors used a 2 T permanent magnet to subject the MH to magnetic stimulations. The results showed that short-term or “acute” stimulation favored the expression of endogenous mechanosensitive ion channel (PIEZO2), allowing the entry of calcium, while long-term, ‘chronic’ application of the stimuli was associated with a decreased expression level of this channel. This study provides evidence that these hydrogels can be used as a research tool for understanding the impact of magnetomechanical stimulation, but may also hold great promise in developing new therapies for reducing mechanosensitive channels expression, well known to cause chronic pain [166]. Moreover, a contemporary investigation has revealed that, through a microscale continuous projection printing technique, a biomimetic spinal cord structure was successfully fabricated; this hydrogel, infused with neural progenitor cells, effectively facilitated axon regeneration within a complete spinal cord injury model in vivo, thereby presenting a promising strategy for the design of magnetically responsive hydrogels intended for neural tissue engineering [167]. In another study, Lacko et al. described a magnetic templating technique to fabricate aligned tubular structures within a hydrogel by using dissolvable magnetic alginate microparticles (MAMs) to form columns under a magnetic field. After removal of these sacrificial MAMs, the scaffolds contained an aligned tubular microarchitecture, which is useful for cell remodeling across different applications. Thus, to mimic the native nerve basal lamina microarchitecture, they used a templated MH made of glycidyl methacrylate, hyaluronic acid, and collagen I. The characterization of this templated MH included assessments of particle alignment and micro-porosity. The removal efficiency of the iron oxide nanoparticles (used at 5 mg/mL) was 97%, whereas the compressive mechanical properties were within the range of peripheral nerve tissue with 0.93 kPa (rat sciatic nerve tissue 1.29 kPa). A preliminary in vivo study for 4 weeks in rats with 10 mm sciatic nerve defect showed that the templated MH guided cellular migration and could aid in peripheral nerve regeneration, inducing moderate improvements in remodeling index and axon density compared with non-templated controls [168]. In addition, Li et al. fabricated electrospun poly lactic-co-glycolic acid (PLGA) fibers containing Fe_3_O_4_ MNPs and cut them by cryo-cutting to obtain magnetic short fibers (MSFs). Then, these MSFs were incorporated into a composite hydrogel of sodium alginate (SA)/carboxymethyl chitosan (CMCS)/multiwall carbon nanotubes (MWCNTs). Under an EMF, the MSFs in the hydrogel aligned and gave rise to magnetic and conductive composite hydrogel MSFs/MWCNTs/SA/CMCS with an oriented morphology. The results showed that MWCNTs improved the electrical and mechanical characteristics of the hydrogel. Furthermore, the alignment of the MSFs in the hydrogel increased its mechanical strength by many folds. In addition, this anisotropic composite hydrogel with magnetic sensitivity and electrical conductivity significantly enhanced the cell viability and proliferation of primary cortical neurons compared to individual magnetic or conductive hydrogels, while providing synergistic effects of electrical and magnetic stimulations to support neurite outgrowth [169]. In another study, Han et al. developed an anisotropic topological chitosan@*Artemisia sphaerocephala* (CS@AS) scaffold (Figure 23) containing Fe_3_O_4_ MNPs modified with dopamine (DFe_3_O_4_, 2 mg/mL) for long-distance peripheral nerve regeneration. The results showed that the anisotropic topological scaffolds, in conjunction with non-invasive wireless magnetic stimulation under an exogenous static magnetic field (SMF), could collaboratively regulate the morphological alterations, proliferation, and directional growth of Schwann cells. In vivo experiments on a rat model of sciatic nerve defect (10 mm) and rabbit model of sciatic nerve defect (35 mm) for 2 and 8 weeks further corroborated that the magnetic CS@AS nerve conduit significantly enhanced the functional repair and reconstruction of long-distance injured sciatic nerves under SMF to a level comparable to that of autograft transplantation. Furthermore, it was established that the scaffolds stimulated the magnetic induction receptors, increased the gene expression for proliferation, migration, and myelin formation, helped to maintain the balance of intracellular calcium concentration, and improved cGMP-PKG, VEGF, MAP Kinase, and TNF signaling [170].

### 4.2. Applications of MHs in Cartilage Tissue Engineering

**Figure 24:**
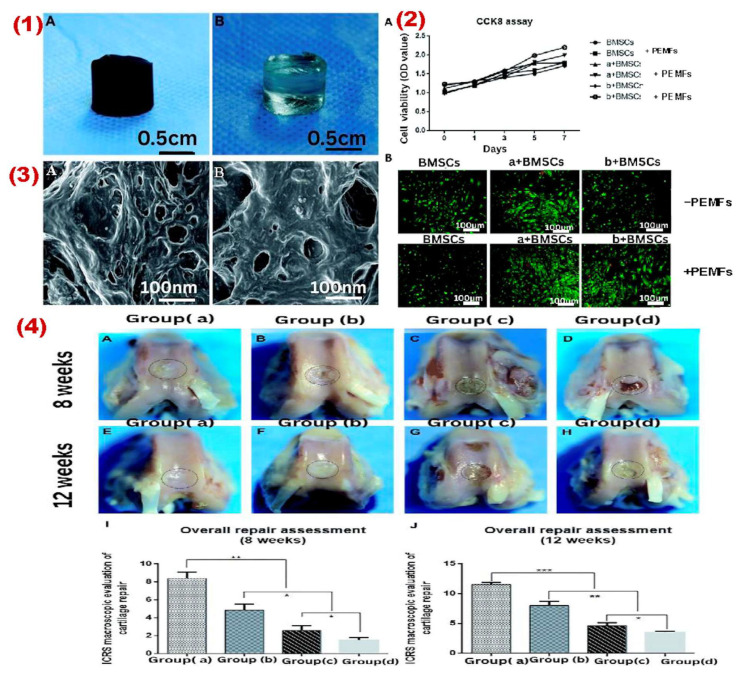
(**1**) Photos of the fabricated hydrogels, (**1A**) magnetic gelatin/β-CD/Fe_3_O_4_ hydrogel, and (**1B**) gelatin/β-CD hydrogel. (**2**) Primary rabbit bone marrow mesenchymal stem cell (BMSCs) proliferation when cultured on the non-magnetic (a) and magnetic (b) hydrogels, evaluated with CCK8 assay (**2A**). PEMFs means that the hydrogel was magnetically stimulated with pulse electromagnetic field. (**2B**) Cell viability evaluated with a live (green fluorescence)/dead (red fluorescence) assay (**3**) Surface characteristics of the hydrogels using SEM. (**3A**) SEM image of magnetic gelatin/β-CD/Fe_3_O_4_ hydrogel; the surface of the material is rough with the presence of pores. (**3B**) SEM image of gelatin/β-CD hydrogel; the surface of the material is quite smooth with small pores. (**4**) The macroscopic characteristics of repaired articular cartilage. (**A**–**H**) Photos of rabbit knee articular defects at 8 and 12 weeks after operation. The black circles show the initial defect margin. (**I**,**J**) Summarize data distribution of the ICRS macroscopic scores of the repaired cartilage at 8- and 12-weeks post-surgery. Data are expressed as mean ± SD; (*n* = 6); * *p* < 0.05, ** *p* < 0.01, *** *p* < 0.001. Group (**a**) magnetic gelatin/β-CD/Fe_3_O_4_ hydrogel with BMSCs (plus pulsed electromagnetic field), Group (**b**) magnetic gelatin/β-CD/Fe_3_O_4_ hydrogel with BMSCs, Group (**c**) magnetic gelatin/β-CD/Fe_3_O_4_ hydrogel, Group (**d**) blank control. Reproduced with permission from [121]. Copyright 2020, The Royal Society of Chemistry (open access).

Magnetic nanoparticles and MHs are known as prospective materials in the field of cartilage tissue engineering due to their effects on stem cells’ behavior and chondrocytes’ response. Another factor that makes MHs favored is the opportunity to deliver drugs, such as growth factors, in a targeted and controlled manner to the area of chondral lesions [171]. Thus, Z. Chen et al. designed a composite hydrogel system with ultrasmall superparamagnetic iron oxide (USPIO)-labeled cellulose nanocrystal (CNC) and silk fibroin (SF) to monitor the degradation of the hydrogel and cartilage regeneration in real time by MRI. Bone marrow mesenchymal stem cells (BMSCs) were loaded in the hydrogel and chondrogenesis was evaluated in vitro, whereas cartilage regeneration was evaluated in vivo in a rabbit cartilage defect model. The results showed that USPIO provided good MR contrast enhancement, allowing for the monitoring of hydrogel degradation. Furthermore, BMSCs proliferated and differentiated well into chondrocytes in the hydrogel. Moreover, an in vivo experiment showed that a smooth neocartilage was observed in the BMSCs hydrogel group at 12 weeks post-operation. Based on these results, the role of the USPIO-labeled CNC/SF hydrogel represents a novel strategy in the field of cartilage tissue engineering. This system is promising for enhancing the repair and regeneration of cartilage, as it promotes chondrogenesis and provides a non-invasive method for monitoring hydrogel degradation [172]. Similarly in another study, Yang et al. grafted KGN onto the surface of USPIO nanoparticles, which formed a nanocarrier system capable of releasing KGN in a slow and controlled fashion at the cartilage repair site. To enhance the efficacy of cartilage repair, a USPIO-KGN nanocarrier was encapsulated within a cellulose nanocrystal/dextran hydrogel, serving both as a delivery vehicle for USPIO-KGN and as a scaffold to foster cartilage regeneration. Both in vitro and in vivo study on a defect rabbit model showed that this methodology provides sustained release of KGN, which promotes the differentiation of BMSCs into chondrocytes. Furthermore, the use of USPIO allowed for non-invasive MRI monitoring of hydrogel degradation and neocartilage generation in vivo consistently with histological analysis [173]. Moreover, Zlonick et al. developed a magneto-patterning technique to position objects (like MSCs, polystyrene beads, PLGA microcapsules) in a hydrogel precursor solution (1% *w*/*v* methacrylated hyaluronic acid (MeHA) with gadodiamide (Gd, 0.2 M) and a photoinitiator (0.5% *w*/*v* lithium phenyl-2,4,6-trimethylbenzoylphosphinate (LAP)). After exposure to a magnetic field (permanent magnet 13,200 Gauss) for 2, 5, or 10 min, the hydrogel was crosslinked under UV (365 nm, 10 mW/cm^2^, 9 min) to fix the objects. Then, the gel was washed to remove the Gd and used for analysis or put in culture medium for cell culture. The results showed that small objects (radius ≤ 1 μm) did not move, while larger objects (5–15 μm) ascended toward the surface of the hydrogel solution. The technique was used for cartilage regeneration by magnetically patterning MSCs with 5 min exposure to the magnet, and then the cells were cultured for 3 to 6 weeks in chondrogenic medium. The analysis showed a cellular gradient (from top to bottom) and the formation of cartilaginous matrix with a gradient of glycosaminoglycan (GAG) [174]. In another study, Huang et al. studied the combined effect of hydrogel material with pulse electromagnetic fields (PEMFs). Figure 24 shows that PEMFs promoted the repair of defective articular cartilage. Thus, they fabricated a gelatin/β-cyclodextrin (β-CD)/Fe_3_O_4_ MNPs hydrogel, and its characterization revealed good mechanical properties (compressive modulus 2.79 MPa) with a microporous surface and unevenness that promote cell adhesion and growth. The result of infrared spectroscopy analysis revealed that the MNPs were uniformly dispersed into the hydrogel, which had reasonably good super paramagnetic characteristics. In addition, when bone marrow stem cells (BMSCs) were cultured on the hydrogel and PEMFs were applied, the expression of cartilage specific gene markers (COL1, COL2, and Aggrecan) was significantly enhanced and BMSCs differentiated into chondrocytes. The hydrogel was also tested in vivo in a knee-defect rabbit model for 8 and 12 weeks. The following four groups were tested: group A (gelatin/β-CD)/Fe_3_O_4_ with BMSCs and PEMFs), group B (gelatin/β-CD)/Fe_3_O_4_ with BMSCs), group C (gelatin/β-CD)/Fe_3_O_4_), and group D (no treatment). The results showed that group A had the best cartilage repair at 8 weeks, and at 12 weeks, the defect was completely filled with new cartilage and restored. In group B, the defect was restored with new cartilage at 50% and 70% at week 8 and week 12, respectively. Therefore, PEMFs combined with MHs promoted cartilage repair. In group C, the regenerated tissue was fibrous, with a gap between the new tissue and the host at week 8, while at week 12, the regenerated tissue was mainly fibrous hyperproliferative tissue with only few cartilage tissues. In group D, there were cavities and fibrous tissue at week 8, while at week 12, there was only inflammatory tissue [121]. Furthermore, Choi et al. fabricated a ferrogel of oxidized hyaluronate (OHA)/glycol chitosan (GC)/adipic acid dihydrazide (ADH)/SPIONs with 3D printing. Mouse teratocarcinoma ATDC5 cells were encapsulated in the ferrogel for chondrocyte differentiation, and a magnetic field was applied. The results showed that the application of this magnetic stimulation enhanced the expression level of the genes SOX-9 and COL-2, proving that the ferrogel has potential for cartilage tissue engineering [175].

### 4.3. Applications of MHs in Bone Tissue Engineering

Bones are the hard tissues which form the skeletal system of vertebrates and contain bone marrow. The ECM of bones is a complex nanocomposite material with periodic hydroxyapatite nanocrystals disordered along collagen fibers, which form hierarchically structured composites from the nano- to macro- levels [176]. Bone cells (e.g., osteoblasts, osteoclasts, osteocytes) are embedded between the collagen fibers and regulate bone turnover (bone growth, bone resorption) along with external stimuli. Conveniently, bones are divided into the following two types: the cortical bone and the trabecular bone. Cortical bones are dense with low porosity (<20%) and are composed of cylindrical structures called osteons or Haversian systems, which are cylinders enclosing a Chandler’s indexer blood vessel surrounded by concentric rings of bone matrix. Trabecular bones or cancellous bones are porous and spongy (>90% porosity), in the form of plates referred to as trabeculae, and have a large surface [177].

**Figure 25:**
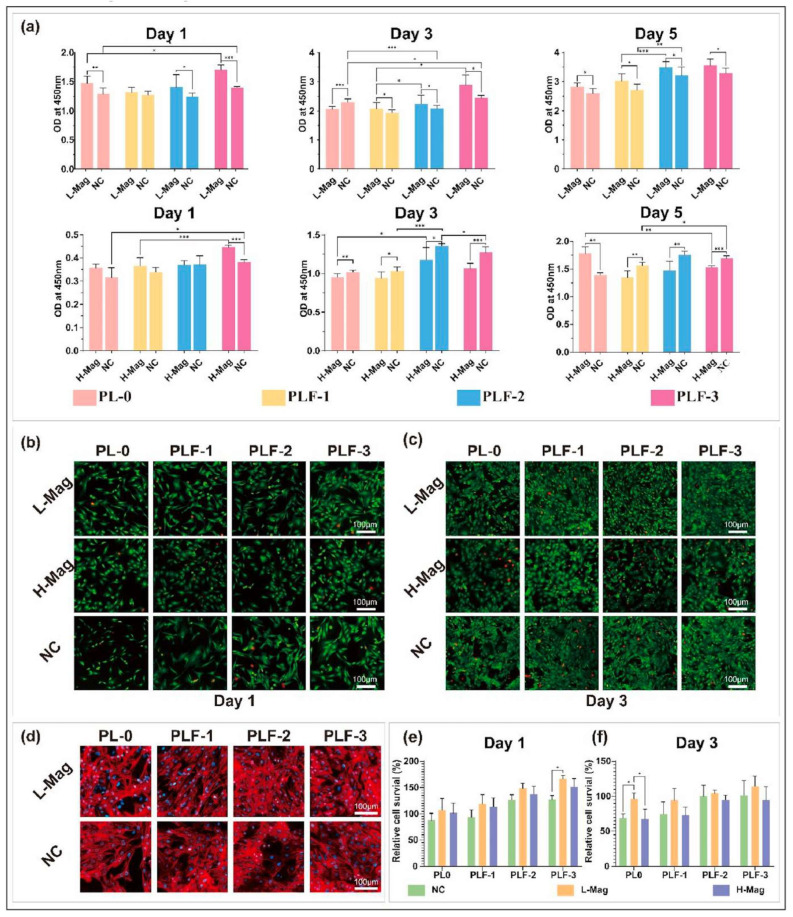
(**a**) CCK-8 assay was used to determine the BMSCs cytotoxicity and proliferation after seeding the scaffolds under H-Mag, L-Mag, and NC for 1 day, 3 days, and 5 days (* *p* < 0.05, ** *p* < 0.01, *** *p* < 0.001). (**b**,**c**) Representative images of fluorescent staining of live (green) and death (red) BMSCs cultured on PL-0, PLF-1, PLF-2, and PLF-3 scaffolds treated with L-Mag, H-Mag, and NC for 4 h (Magnification ×200). (**d**) The shape of the BMSCs when they were cultured on the surface of the scaffolds using L-Mag and NC for 24 h. DAPI was used to stain the nucleus, which appears blue, while F-actin was stained using TRITC and appears red (scale bar 100 μm). (**e**,**f**) Quantification of living cells using a live/dead staining of BMSCs (* *p* < 0.05). Reprinted with permission from [179]. Copyright 2024, The Authors (open access).

Magnetic materials (MNPs, MHs) have been used with magnetic fields for the benefits of bone tissue engineering applications. Thus, Arjmand et al. showed that the osteogenic differentiation of adipose-derived mesenchymal stem cells (ADSCs) cultured on a PCL scaffold improved in the presence of PEMF [178]. It is known that magnetic stimuli acting at the interfacial level trigger sensitive receptors on the cell membrane, leading to an increase in metabolic activity. In a recent study, Xu et al. fabricated composite scaffolds of PLA, PCL, and Fe_3_O_4_ nanoparticles by a microinjection molding technique. Depending on their magnetite weight ratio (7/3/0,1,2,3) these PLA/PCL/Fe_3_O_4_ scaffolds were named PL-0, PLF-1, PLF-2, and PLF-3. These scaffolds showed excellent physicochemical properties and biocompatibility. Furthermore, the PLA/PCL/Fe_3_O_4_ scaffolds accelerated the adhesion and proliferation of bone mesenchymal stem cells (BMSCs) under a relatively weak magnetic field (25–30 mT). Thus, the BMSC proliferation on scaffolds PL-0, PLF-1, PLF-2, and PLF-3 was characterized for five consecutive days with various static magnetic fields (SMFs) applied. The results showed that an increase in Fe_3_O_4_ in the scaffold structure enhanced the cellular response and that this was in sync with the assay of cytotoxicity (Figure 25a,e). However, with L-Mag (25–30 mT), the PLF scaffolds had a positive effect on cell proliferation, while with H-Mag (92–102 mT), the PLF scaffolds had a negative effect on cell proliferation (Figure 25a), consistent with the quantitative analysis of live/dead staining (Figure 25e,f). The amounts of cells grown on the PLF scaffolds in the magnetic culture groups compared to the NC group (<0.05 mT, earth magnetic field) were significantly enhanced at day 3 and day 5 of culture (Figure 25a), excepted for the PL-0 scaffold on day 3 of culture [179]. In another study, Babakhani et al. developed a nanocomposite scaffold made of polyvinyl alcohol (PVA)-chitosan (CS), modified clay (C30B) with graphene oxide (GO), and hydroxyapatite (HAp) to improve the mechanical characteristics of the hydrogel. Different scaffolds were investigated using FT-IR, XRD, SEM, TGA, swelling rate, degradation tests, and evaluation of biological and mechanical properties. The analysis proved that the addition of 2% of clay-modified graphene oxide with magnetite C30B/GO/Fe_3_O_4_ enhanced the compressive strength of the polymeric material (PVA/CS) by 23 times. In addition, the combination of HAp and modified clay increased the mineralization levels on the surface of the scaffold. Thus, the prepared nanocomposite scaffold had a compressive strength of 9.31 MPa, a porosity of 75%, and a pore size of about 50 nm, like a cancellous bone. The swelling behavior was in accordance with the desired characteristics for bone scaffolds, with a swelling amount of 1790%. The cytotoxicity studies showed that all prepared scaffolds were biocompatible with good cell viability. The study concluded that PVA/CS/C30B/GO/Fe_3_O_4_ had good mechanical properties and can be used in bone tissue engineering [180].

### 4.4. Application of MHs in Cardiac Tissue Engineering

Cardiac tissue engineering requires the use of matrices that mimic the physical, spatial, and electrical signals seen in the natural ECM [181]. Consequently, scaffolds for cardiac tissue engineering should mimic the native cardiac tissue elasticity, direct the alignment and elongation of cardiomyocytes, and have some electrical conductance to favor their contraction [182]. To this end, conductive polymers such as polyaniline [70,183] or polypyrrole [184], as well as with the incorporation of noble metals [185], carbon nanotubes (CNTs, MWNTs), or carbon-based particles [37,38,71,186], have been used to improve the polymer conductivities and have been applied for skeletal muscle and cardiac muscle tissue engineering. Regarding the latter, the rod-shape morphology of carbon nanotubes for cardiac tissue engineering applications makes them especially suited. Such types of CNTs have been used to generate well-defined faceted substrates with abundant porosity, which enables the alignment of the cells and ECM deposition alongside the fabricated features to replicate the organization of cardiac native tissues [187,188].

**Figure 26:**
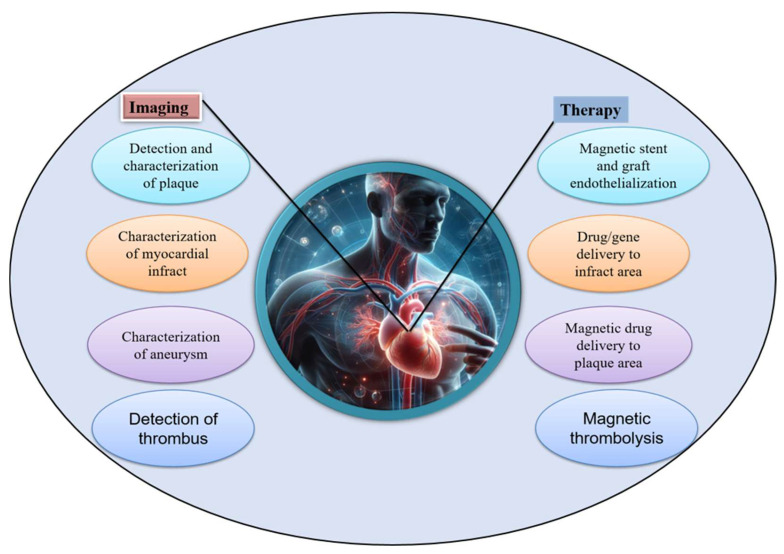
Diverse uses of iron oxide nanoparticles that have been approved for use in cardiovascular imaging and treatment. Modified with permission from [195]. Copyright 2020, Elsevier B.V.

Similarly, Bonfrate et al. used external magnetostatics fields derived from two parallel magnets or current wires to micropattern paramagnetic iron oxide nanoparticles (MNPs) in collagen matrices used as a scaffold for cardiac tissue engineering. By using external magnetostatics fields derived from two parallel magnet or current wires, it was possible to micropattern the MNPs in the collagen matrices and to increase the electrical conductivity of the designed scaffolds [189]. In another study, a bilayer of PEGDA with different molecular weights was self-assembled in 2D, functionalized with commercially available MNPs, and used for cardiac and muscle tissue engineering. The study showed that the PEGDA layers with defined thickness, stiffness, and swelling could rolled to form a 3D tube loaded with cardiomyocytes under a 150 mT magnetic field [190]. Furthermore, Liu et al. fabricated injectable magnetically responsive cryogels based on GelMA and methacrylate elastin containing CNTs and Mn_3_O_4_ MNPs for potential use in cardiac tissue engineering and evaluated them under compression tests and shape memory tests [191]. In addition, Han et al. loaded cardiomyocytes H9C2 with SPIONs, which significantly enhanced their expression of connexin 43 (Cx43), which is a gap junction protein. Then, they used these H9C2-SPIONs to prime MSCs in coculture via active cellular crosstalk, which induced MSCs with a cardiac-repair-favorable paracrine profile, enhancing the therapeutic efficacy of MSCs in the frame of myocardial infarction [192]. Kankala et al. wrote a review on the nanosize components involved in cardiac tissue repair and on the nanomaterials that are used to mimic these features. Thus, nanoparticles have been used alone or loaded into hydrogels or other biomimetic constructs to impart the structural and electrical reorganization of native cardiac tissue to achieve better regenerative outcomes [193]. In another study, SPIONs (1 wt%) modified with casein were incorporated into electrospun silk fibroin nanofibers to enhance the mechanical properties and surface characteristics of the scaffolds. The characterization of the fabricated SF nanofibers containing SPIONs was performed using SEM, TEM, water contact angle, and tensile tests. The nanofibrous scaffold was used as a substrate for culturing mouse embryonic cardiac cells (ECCs), and the results showed that ECCs had better attachment, elongation, alignment and density on SF–SPIONs–casein compared to SF only. Moreover, the immunostaining analysis at day 7 of culture showed an enhanced expression of mature cardiac markers such as α-MHC and c-TnT in ECCs cultured on SF–SPIONs–casein compared to those cultured on SF nanofibrous scaffolds. In addition, real-time PCR analysis indicated the upregulation of functional genes involved in cardiac muscularity, including GATA-4, cardiac troponin, Nkx 2.5, and α-MHC for ECCs cultured on SF/SPION/casein scaffolds compared to ECCs cultured on SF scaffolds only [194]. Therefore, nanoparticles are important components for cardiac tissue engineering, and there have been many advancements in the use of MNPs both for diagnosis and treatment of cardiovascular diseases, as shown in Figure 26. Especially, iron oxide-based MNPs have been described in detail as nanosized agents with superparamagnetic properties used for therapy in the frame of cardiac vascular diseases and also used for imaging and the visualization of plaques, thrombus, drug delivery, new reendothelialization of stents, and blood vessel regeneration [195].

In the following, we give a table (Table 2) showing the different MHs used in tissue engineering applications.

## 5. Conclusions

As we have seen in this review, MHs are composed of a hydrogel matrix with encapsulated magnetic particles. Therefore, they have the properties of hydrogels with the additional properties of magnetic particles. Hydrogels are used in a wide variety of biomedical applications and are one of the major biomaterials used due to their viscoelastic property that mimic natural tissues and their biocompatibility. Magnetic particles are nano-, micro-, or millimeter-sized particles of different compositions, including iron oxide, nickel, and cobalt, that are sensitive to magnetic fields. They bring to the hydrogel properties of actuation, speed, and remote control. Their use in isotropic and anisotropic MHs’ fabrication has induced the development of different methods of fabrication, and these MHs have begun to be involved in a growing number of varied applications. As shown in this review, we have highlighted a large number of these applications in the biomedical field such as hyperthermia, drug delivery, wound healing, MRI, detection with sensors, and tissue engineering (neural, cartilage, bone, cardiac tissues). The use of MHs in these different domains has generated major developments, and the research field with MHs is very dynamic.

However, although MH developments are already significant, several aspects need to be further explored before their use in clinic or their commercialization. Thus, the toxicity of MNPs is an important topic and has been evaluated. The results showed that their toxicity depends on their size, with greater toxicity for a bigger particle size. Therefore, the use of magnetic nanoparticles is favorable and they are easily excreted from the body. However, biological tissues have different sensitivities to chemicals and materials and, therefore, MNPs’ toxicity should be evaluated following their targeted application, and with potential long-term application. The concentration of MNPs used in MHs is usually relatively low, and the use of higher concentrations may be explored. Likewise, the use of other materials with a strong magnetic response may be explored for improved functionalities, improved control over the engineered tissue construct, and to decrease the amplitude of the magnetic field required for the manipulation of MHs. Otherwise, different improvements are required depending on the targeted application. For example, hyperthermia improvements can be made to obtain more heat with a more precise location to enhance the efficacy of treatment. In drug delivery with MHs, accurate controlled release is needed, and the new development trend is toward pulsatile drug release. For medical imaging, MRI with contrast agents is very efficient for providing deep penetration and clarity. However, its slow acquisition time has been a major drawback that was overcome with more recent apparatus, including deep learning image reconstruction, super resolution algorithms, and the use of parallel imaging, multi-slice imaging, and compressed sensing. However, implementing these accelerated MRI protocols in clinics enhances the cost and workload of specialists. In tissue engineering, more improvements are possible for the fabrication of efficient MHs that combine controlled magnetic properties with the required mechanical properties and biological performance. The design of MHs with precise anisotropic magnetic patterns is not straightforward. Furthermore, the development of magnetically graded hydrogels may be interesting.

Nowadays, with the current technological advances, the use of additive manufacturing techniques such as direct ink writing (DIW), stereolithography, and 3D printing boosts the development of new MHs, allowing for more complex structure formation, fabrication of MHs with multi-materials, patient-specific tailoring, automation, and reduced fabrication time. Another new trend for the development of MHs is in the integration of multi-stimuli with magnetic actuation. Furthermore, the development of magnetic micro/nanorobots that can be remotely activated is another axis of MH development. Moreover, with the development of artificial intelligence, further advances in MH fabrication are possible. The diversity of application of MHs is already important, and cross-disciplinary cooperation among different fields (e.g., material science, advanced manufacturing, biomedical engineering) will further enhance their development.

## Figures and Tables

**Table 1:** Advantages and disadvantages of some fabrication strategies for MHs.

Strategy	How It Works (for Magnetic Gels)	Advantages	Disadvantages	Applications	Ref.
In situ gelation	Gel forms directly at target site (pH, temperature, ionic, or light-responsive) with embedded magnetic nanoparticles.	Minimally invasive, injectable, conforms to defects, easy drug loading.	MNP dispersion control is difficult, risk of burst release, uniformity issues.	Injectable depots, localized hyperthermia, image-guided therapies.	[59,60]
Freeze-drying (cryogels/aerogels)	Polymer-MNPs slurry frozen; ice templating + sublimation gives porous, magnetic scaffold.	Very high porosity, good permeability, tunable pore size, easy drug loading	Brittle, variable mechanics, sterilization issues, possible MNPs shedding.	Bone/tissue scaffolds, wound dressings, on-demand thermal release.	[61]
Electrospinning	Electric field spins MNPs-loaded polymer jets into nanofibers/fiber mats.	High surface area, anisotropy, tunable alignment, good for directional cell growth.	Fabrication is often confined to thin layers, with concerns over solvent biocompatibility and aggregation of magnetic nanoparticles affecting uniformity.	Nerve/muscle/ cartilage scaffolds, guided regeneration.	[62,63]
3D bioprinting	Extrusion/DLP of MNPs-laden bioinks into ordered, complex constructs.	Patient-specific structures, gradient/multi-material capability, good cell viability.	Balancing print accuracy with nanoparticle concentration is challenging, and cells may experience shear stress during extrusion.	Custom tissue scaffolds, drug depots, actuators.	[55,64]
Micropatterning	Lithographic or photopatterning techniques generate defined spatial architectures while embedding magnetic domains in targeted regions	Enables high spatial resolution and patterning, compatibility with bioelectronic systems, and delivery of site-specific biological signals	Primarily limited to 2D or thin constructs, with low fabrication throughput and demanding equipment requirements	In vitro models, patterned scaffolds, bioelectronic interfaces.	[65]
Microfluidics	Using flow focusing, microfluidic systems can reliably fabricate magnetic microgels and fibers with controlled and consistent sizes.	Highly uniform particles with superior encapsulation capacity and versatile modular assembly.	Challenges include clogging of devices, restricted surfactant compatibility, and difficulties in large-scale translation.	Injectable microgels, targeted drug delivery, modular tissues.	[66,67]
Field-assisted templating (ordering)	External magnetic field aligns MNPs/fibers during gelation to create anisotropic gels.	Mimics natural anisotropy, improves guidance cues, enhances actuation.	Risk of MNPs clustering; requires specialized hardware.	Cartilage, tendon, neural scaffolds, actuators.	[36]

**Table 2:** Table of magnetic hydrogels (MHs) used in various tissue engineering applications.

Hydrogel	MNPs	Additional Properties	Application
Poly (acrylic acid-*co*-vinyl sulfonic acid) PAAVSA/Fe_3_O_4_ Hydrogel	Fe_3_O_4_ Magnetic nanoparticles (MNPs)	The pH reversibility of the PAAVSA/Fe_3_O_4_ hydrogel was further examined in the views of the swelling/deswelling cycling for the given set of characteristic pH numeric, pH 4.1–7.	pH-responsive hydrogel for healthcare applications including drug delivery systems, diagnosis of diseases, and biosensors [21].
Gelatin methacrylate/Fe_3_O_4_ magnetic hydrogel	Fe_3_O_4_ MNPs (0–15 mg/mL)	Several passive magnetic-based devices have shown potentials in wireless biomechanical monitoring in terms of high sensitivity and non-contact sensing, but these devices suffer from issues with mechanical properties, biocompatibility, and sensitivity contradictions.	The developed GelMA/Fe_3_O_4_ magnetic hydrogels’ mechanical properties are close to natural tissue, and they have a stable sensing capacity for minor strains in ionic solution for long-term monitoring [19].
Oxidized hydroxypropyl cellulose with carboxymethyl chitosan, an injectable hydrogel	Fe_3_O_4_ MNPs (15 mg/mL)	The chemotherapeutic agent Artemisinin (ART) was integrated into the three-dimensional network architecture of the Nanoparticle-hydrogel (NP-hydrogel) to fabricate the ARTNP-hydrogel, facilitating targeted cancer therapeutic interventions.	This composite hydrogel has multiple functions, including magnetic targeting, pH sensitivity, chemodynamic therapy, and photothermal response [196].
Magnetize deacetylated chitin nanofibers (M-DEChNs) hydrogel	Fe_3_O_4_ (0–89.2 mM/L)	This M-DEChN showed cytocompatibility against ATDC-5 cells and could be heated in AMF to kill osteosarcoma in vitro/in vivo by the temperature.	The gel synthesized in the present study demonstrates remolding capacity, biocompatibility, and exhibits antitumor properties, rendering it applicable as a tumoricidal agent or as an adjunctive treatment following tumor resection [197].
Xanthan gum/Fe_3_O_4_-based drug-loaded magnetic nanoparticle composite hydrogel	Fe_3_O_4_ MNPs (0 (*w*/*w*) and 10% (*w*/*w*) of the mass of the polymer)	In addition to an enhanced activity of the drug-loaded hydrogel compared to the free drug, results showed that the application of an alternating magnetic field efficiently stimulated a 3-fold faster release of the encapsulated drug compared to passive conditions, whereas a concentration-dependent shortening of the water protons’ relaxation time at a clinical field of 3 T confirmed this magnetic hydrogel as a *T*_2_-MRI contrast enhancer.	XG/Fe_3_O_4_ magnetic nanoparticle composite hydrogels represent a novel generation of multifunctional theranostic platforms designed for injection and implantation across various clinical contexts, including postoperative applications in oncology, wound healing in dermatology, and in dental practices, among others [136].
GelMA–PVA magnetic (GPM)	IONs (1, 5, and 10% *w*/*v*)	An in vitro cytocompatibility test showed that all formulations were biocompatible and that PTH addition significantly promoted the proliferation of MC3T3-E1 pre-osteoblasts.	This recently formulated GPMP sample facilitates simultaneous osteogenic effects through the controlled release of PTH and magnetically mediated bone regeneration, demonstrating potential in enhancing bone healing and addressing various delayed or non-union conditions without the necessity of daily injections [198].
Magnetic Glycol Chitin-Based Hydrogel	Fe_3_O_4_ MNPs (250–750 μg Fe/mL)	The prepared hydrogel nanocomposite was nontoxic toward HeLa cells when exposed for 2 h, in contrast to similar concentrations of antibiotics used in the clinical setting (i.e., vancomycin).	This pioneering treatment methodology, enabled through the utilization of nanocomposites, exhibits substantial potential for addressing chronic infections associated with bacterial biofilm proliferation, which is often correlated with persistent external wounds in bedridden patients and individuals suffering from chronic diabetic foot conditions [199].
Fmoc(fluorenylmethoxycarbonyl)-RGD (arginine–glycine–aspartic acid)/MNP hydrogel	Fe_3_O_4_ MNPs (0.1 vol % of sample).	In the current investigation, the conjunction of cellular components and magnetic nanoparticles exhibited a synergistic influence in mitigating degradation within magnetic peptide hydrogels.	This research introduces an innovative methodology aimed at enhancing the physical and mechanical characteristics of supramolecular hydrogels through the integration of magnetic nanoparticles, which provide structural reinforcement and stability, enable remote actuation via magnetic fields, and improve injectability [200].
Hybrid hydrogel containing type II collagen, hyaluronic acid (HA), and polyethylene glycol (PEG) and incorporated magnetic nanoparticles hydrogels (collagen II-HA-PEG hydrogel)	MNPs (10 mg/mL)	In addition, the presence of magnetic nanoparticles did not affect the viability of BMSCs within 24 h of culture when compared with the control group.	This investigation presents a promising magnetically responsive nanocomposite hydrogel for prospective applications in cartilage tissue engineering, warranting further examination of its impact on cellular functions when synergistically combined with electromagnetic stimulation [201].
Magnetic-Responsive PVA Hydrogels	MNPs (0.25% to 1% *v*/*v* of PVA solution)	The extent of reversibility in protein sorption–desorption processes was observed to enhance with a reduction in magnetic field intensity to 0.45 Tesla.	The advancement of bioseparation systems characterized by superior performance, specifically those exhibiting decreased susceptibility to biofouling, as well as the design of magnetically controlled drug delivery systems, biosensors, and tissue engineering devices endowed with enhanced efficiency [10].
Magnetic-responsive aligned fibrin hydrogel (MAFG)	Fe_3_O_4_ MNPs (10 mg/mL)	A comparative analysis revealed a diminished cell proliferation rate within the initial three days of culture for the MAFG group relative to the AFG group, while the cell counts for MAFG and AFG after five days of culture exhibited no statistically significant difference.	MAFG@MF facilitates axonal regrowth and promotes functional neuronal regeneration, thereby significantly contributing to the restoration of motor function following spinal cord injury [202].
Alginate-magnetic short nanofibers 3D composite hydrogel	SPIONs 10% *w*/*w* of polymer	The magnetic SNF/hydrogels demonstrated a notably elevated expression of the neuron-like cell marker β-tubulin III in comparison to their non-magnetic counterparts, indicating that the magnetic characteristics of the composite hydrogel can foster neural-like differentiation of Olfactory Epithelial–Mesenchymal Stem Cells (OE-MSCs).	The alginate-magnetic short nanofibers 3D composite hydrogel enhances the bioactivity of encapsulated human olfactory mucosa stem cells, presenting promising prospects for nerve regeneration applications [203].
Magnetic PLGA Microsphere-Gelatin Hydrogel	Fe_3_O_4_ MNPs (200, 400, and 800 mg/L)	GelFe_3_O_4_-400 had the best effect on promoting the growth of pre-osteoblasts under 20 mT static magnetic field in this experiment.	The magnetic poly(lactic-co-glycolic acid) microsphere-gelatin hydrogel exhibits remarkable application potential in promoting osteogenesis and facilitating bone repair [204].
Methacrylate–chondroitin sulfate magnetic nanoparticles (MA-CS MNPs) hydrogel	Fe_3_O_4_ MNPs (2% (*w*/*v*))	The impact of electromagnetic field (EMF) stimulation was also evaluated, revealing its capacity to modulate cellular responses, thereby demonstrating the feasibility of generating gradient tissue constructs through magnetic responsive hydrogels.	The proposed hydrogel system facilitated the development of a tendon-to-bone interface model for the investigation of cellular crosstalk [205].
Silk Fibroin hydrogel-loaded Fe_3_O_4_@PAA NPs	Fe_3_O_4_ NPs(0.8 mg/mL)	Fe_3_O_4_@PAA Silk Fibroin (SF) hydrogel exhibits hydrogen peroxide scavenging activity.	Silk fibroin hydrogel incorporated with Fe_3_O_4_@PAA nanoparticles in a static magnetic field environment promotes osteogenic differentiation [206].
RSF/TA/Fe_3_O_4_ Hydrogel	Fe_3_O_4_ (1%–5% *w*/*v*)	The RSF/TA/Fe_3_O_4_ hydrogel demonstrates adequate adhesion within biological microenvironments and exhibits a robust osteogenic effect both in vitro and in vivo when subjected to an external static magnetic field (SMF), thereby rendering it applicable for the repair of critical-sized bone defects.	A methodical approach for the development of a rapid-gelling, shape-adaptive, highly adhesive, and magnetically responsive nanocomposite hydrogel via the precipitation technique has been established, presenting a promising biomaterial for tissue engineering aimed at facilitating the repair of irregular bone defects in the foreseeable future [207].
Alginate/poly-l-ornithine/gelatin (alginate-PLO-gelatin) hydrogel	Fe_3_O_4_ (16.67 μg/mL)	The differentiation of endothelial progenitor cells (EPCs) into endothelial cells was substantiated, and their capacity to secrete pro-angiogenic growth factors was found to significantly enhance both cell migration and vascularization.	The augmented regeneration of blood vessels in the injured region through the administration of EPCs affixed to the hydrogel sheet indicates that the proposed system possesses substantial potential as a therapeutic modality for tissue regeneration [78].
k-Carrageenan based magnetic@polyelectrolyte complex composite hydrogel	FeNP (0.05 wt% of total volume of PHMG)	Under the synergistic conditions of pH and temperature stimuli (pH 5.0/42 °C), the formulated hydrogel system exhibited remarkable drug loading efficacy (~ 68%) alongside improved drug release characteristics.	The magnetic polyelectrolyte complex-based hydrogel (MPEC) is deemed appropriate for application in pH- and temperature-responsive controlled drug delivery systems pertinent to cancer therapy [208].
Tragacanth-silk fibroin hydrogel (TG/SF/Fe_3_O_4_)	Fe_3_O_4_	The efficacy of hyperthermia using the hybrid (TG/SF/Fe_3_O_4_) scaffold was assessed, revealing a maximum specific absorption rate (SAR) value of 41.2 W/g recorded during the initial interval.	The resulting TG hydrogel/SF hybrid was magnetized with Fe_3_O_4_ MNPs for hyperthermia application [209].
Pluronic thermoresponsive hydrogel	SPIONs 5 mg/mL	The developed hydrogel/microparticle system demonstrated a lower drug release rate compared to the microparticles utilized in isolation.	The Pluronic thermoresponsive hydrogel represents a viable thermoresponsive drug delivery system (DDS) suitable for magnetic hyperthermia applications, thereby facilitating a synergistic approach to cancer treatment [210].
Salecan-g-PCH/Fe_3_O_4_@SiO_2_ composite hydrogels	Fe_3_O_4_@SiO_2_ nanoparticles (2%, *w*/*v*)		Salecan-g-PCH/Fe_3_O_4_@SiO_2_ composite hydrogels exhibit potential as carriers for anticancer drugs, particularly within the context of magnetically targeted drug delivery applications [211].

## Abbreviation

The following abbreviations are used in this manuscript:

AA	Acrylamide
AMF	alternating magnetic field
CNS	central nervous system
CS	Chitosan
DOX	Doxorubicin
EPC(s)	endothelial progenitor cell(s)
ECM	extracellular matrix
GelMA	gelatin methacrylate
GOx	glucose oxidase
LCST	low critical solution temperature
MH(s)	magnetic hydrogel(s)
MHT	magnetic hyperthermia therapy
MNPs	magnetic nanoparticles
MRI	magnetic resonance imaging
MSC(s)	mesenchymal stem cell(s)
MWCNTs	multi-walled carbon nanotubes
PCL	polycaprolactone
PEG	poly(ethylene glycol)
PGA	poly(glycolic acid)
PLGA	poly(lactic-co-glycolic) acid
PNIPAM	poly(N-isopropylacrylamide)
PVA	polyvinyl alcohol
SF	silk fibroin
SMC(s)	smooth muscle cell(s)
SPIONs	superparamagnetic iron oxide nanoparticles
SWCNTs	single-walled carbon nanotubes
VSA	vinyl sulfonic acid
